# Mixed Phenanthroline–Bipyridine Europium and Ytterbium Cryptates—Bright Luminophores with Small Radiative Lifetimes

**DOI:** 10.1021/acs.inorgchem.5c05828

**Published:** 2026-02-22

**Authors:** Tobias Haas, Timo Neumann, Nicholas Jobbitt, Xiaoyu Yang, Hartmut Schubert, David Hunger, Michael Seitz

**Affiliations:** † Institute of Inorganic Chemistry, University of Tübingen, Auf der Morgenstelle 18, 72076 Tübingen, Germany; ‡ Institute of Physics (PHI), Karlsruhe Institute of Technology, 76131 Karlsruhe, Germany; § Institute for Quantum Materials and Technologies (IQMT), Karlsruhe Institute of Technology, 76131 Karlsruhe, Germany

## Abstract

New phenanthroline-based tris­(biaryl) europium­(III) and ytterbium­(III) cryptates with great luminescence efficiencies in solution are reported. 1,10-Phenantroline, in combination with 2,2′-bipyridine-*N*,*N*′-dioxide, delivers cryptates with high rigidity, efficient lanthanoid­(III) sensitization (η_sens_) and small radiative lifetimes τ_rad_ for Eu and Yb. The powerful synergy of both bidentate chelating units results in excellent absolute quantum yields of up to 20% for the europium complexes in aqueous solution, which indicates an impressive improvement compared to other well-known tris­(bipyridyl)-based Eu­(III) cryptates.

## Graphical abstract

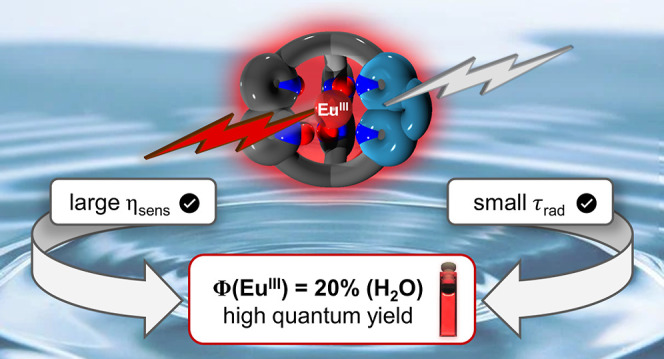

## Introduction

Photoluminescence in molecular lanthanoid complexes has found a wide array of fascinating applications due to their unique properties over the last decades.^1^ While most of the processes affecting lanthanoid luminescence (such as energy transfer from the ligand to the metal or intramolecular quenching of excited states) have been investigated and optimized thoroughly, the radiative luminescence lifetime τ_rad_ is still not completely understood.^2^ In contrast to the measured (observed) luminescence lifetime τ_obs_, the radiative lifetime τ_rad_ of an excited state is the hypothetical luminescence lifetime in the absence of any other nonradiative deactivating processes. Little attention has been paid to this key parameter over the last decades, despite its important role in determining the absolute quantum yield *Q*_Ln_^L^ as can be seen in eq 1

1 $$Q_{\mathrm{Ln}}^{L}=\eta_{sens}\cdot Q_{\mathrm{Ln}}^{\mathrm{Ln}}=\eta_{sens}\cdot \frac{\tau_{obs}}{\tau_{rad}}$$

The absolute quantum yield is determined by the sensitization efficiency η_sens_ (including intersystem crossing and energy transfer from the ligand to the lanthanoid) and by the intrinsic quantum yield *Q*_Ln_^Ln^. The intrinsic quantum yield is equal to the ratio of the observed radiative rate constant *k*_r_ (=1/τ_rad_) and the nonradiative deactivation rate constant *k*_nr_ according to eq 2

2 $$Q_{\mathrm{Ln}}^{\mathrm{Ln}}=\frac{k_{r}}{k_{r}+k_{nr}}$$

eqs 1 and 2 make clear that increasing the overall quantum yield *Q*_Ln_^L^ is dependent on the simultaneous optimization of a whole set of parameters (η_sens_, *k*_r_ = 1/τ_rad_, *k*_nr_). Nevertheless, when hypothetically leaving all factors other than τ_rad_ constant, decreasing the radiative lifetime makes the emission more competitive compared to nonradiative deactivation processes which leads to higher emission efficiencies. It should be pointed out that the total light intensity that can be detected does not depend solely on the absolute quantum yield *Q*_Ln_^L^ but instead should be evaluated using the brightness *B* (*B* = *Q*_Ln_^L^·ε), which also takes into account the molar extinction coefficient ε at the excitation wavelength. In addition, the question if a particular application benefits from short (e.g., most OLED applications) or long (e.g., time-gated luminescence bioassays) observable luminescence lifetimes τ_obs_ is also connected to whether short or long intrinsic lifetimes τ_rad_ are advantageous.

Since the valence f-orbitals in trivalent lanthanoids are shielded from covalent interactions with the surrounding by filled 5s/5p-orbitals, f–f-transitions are relatively strictly Laporte-forbidden. In contrast to e.g. transition metal complexes, here this selection rule cannot easily be relaxed by admixture of states/vibrations of opposite parity. Therefore, influencing τ_rad_ in Ln^3+^ ions is not straightforward and our knowledge in this area is quite fragmentary.^2^ There are still only very few examples in the literature where changes in radiative lifetimes can be assigned unambiguously to modifications of the coordination sphere of the lanthanoid ion instead of depending on a completely different donor set.

Our interest in this subject was initiated by investigations of Bünzli et al. that the bidentate ligand 2,2′-bipyridine-*N,N*′-dioxide is able to decrease the radiative lifetime in europium­(III) β-diketonate complexes substantially.^3^ Related work by us later confirmed an analogous effect for tris­(bipyridine)-based cryptates of Yb, Eu, and Nd (Figure 1).^4,5^

**Figure 1:**
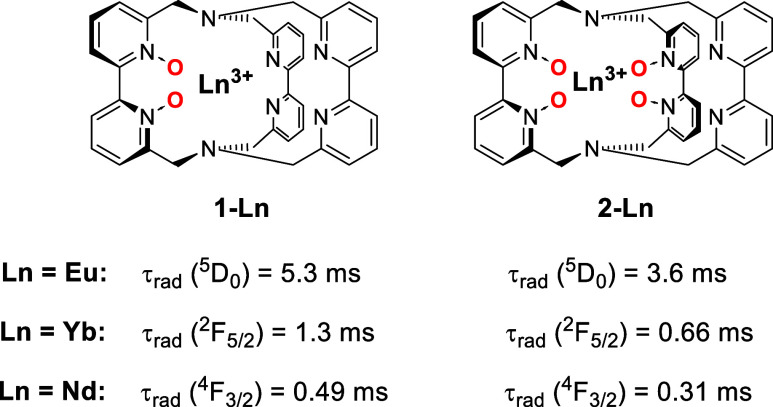
Tris­(bipyridine)-based lanthanoid cryptates **1-Ln** and **2-Ln** with different contents of pyridine-*N*-oxide moieties and their influence on τ_rad_.^4,5^

In the studies on europium^5^ and ytterbium^4^ we showed that more pyridine-*N*-oxide binding motifs in cryptates are responsible for an increased rate of emission. Concomitantly, however, the 2,2′-bipyridine-*N,N*′-dioxide units unfortunately had a negative influence on sensitization efficiencies η_sens_, which inevitably led to reduced absolute quantum yields, especially in the case for europium.^5^ This unwanted effect presumably resulted from the introduction of low-lying CT states through higher *N*-oxide contents, which seems plausible for europium and ytterbium, both of them having accessible divalent oxidation states.^6^ In an effort to alleviate this problem, we turned our attention to other reports in the literature that 1,10-phenanthroline has a similar impact compared to pyridine-*N*-oxides for reducing radiative lifetimes in luminescent europium complexes. For example, τ_rad_ of the europium­(III) β-diketonate complex **4-Eu** using 1,10-phenanthroline as ancillary ligand is 23% smaller compared to its 2,2′-bipyridine analogue **3-Eu** (see Figure 2).^7^ In a second example, it was shown in the literature that 1,10-phenanthroline is responsible for an efficient energy transfer in europium­(III) nitrate complex [Eu­(phen)_2_(NO_3_)_3_] (η_sens_ = 72%).^8^ Based on the previous literature precedence, 1,10-phenanthroline seemed to us in this context to be an attractive photosensitizer in addition to its generally beneficial properties of also being much more rigid than 2,2′-bipyridine.^9^

**Figure 2:**
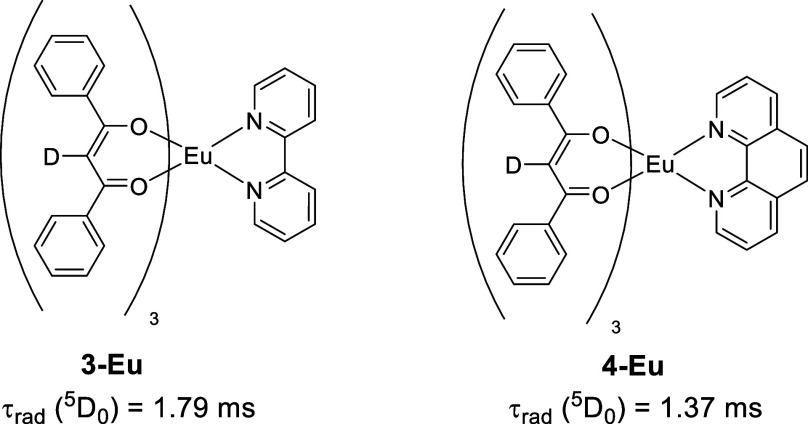
Europium β-diketonate complexes **3-Eu** and **4-Eu** using 2,2′-bipyridine and 1,10-phenanthroline as ancillary ligands, respectively.^6^

Here, we expand our previous study for europium and ytterbium tris­(bipyridine) cryptates^4,5^ by combining 2,2′-bipyridine-*N,N*′-dioxide motifs with 1,10-phenanthroline in cryptate scaffolds in order to achieve further insights into how the radiative lifetime τ_rad_ can be utilized in a systematic manner to modulate the luminescence in these lanthanoid complexes. We also exclusively focus on the pure photophysics in order to enhance the overall quantum yield *Q*_Ln_^L^ without taking into account practical considerations such as brightness or application-specific requirements.

For this purpose, we designed the two novel cryptates **5-Ln** and **6-Ln** (Figure 3) which are very similar to the tris­(2,2′-bipyridine)-based cryptates **1-Ln** and **2-Ln** (Figure 1). The former share as many features as possible and only differ in the number of (a) *N*-oxide donor moieties and (b) phenanthroline motifs. Moreover, all mentioned cryptates (**1-Ln**, **2-Ln**, **5-Ln**, **6-Ln**) exhibit *C*_2_-symmetric structures in solution. The similarities among all tris­(bipyridine) cryptates provide a perfect setup for the interpretation of the changes in radiative lifetimes τ_rad_ and sensitization efficiencies η_sens_. For the Yb cryptates, where strong multiphonon quenching of the metal-centered, excited states by ligand C–H oscillators can be expected,^10^ we also prepared the partially deuterated cryptate **[D**_**8**_ **]-6-Yb** (Figure 3), in order to also study the effect that deuteration has on the improvement of luminescence efficiencies.

**Figure 3:**
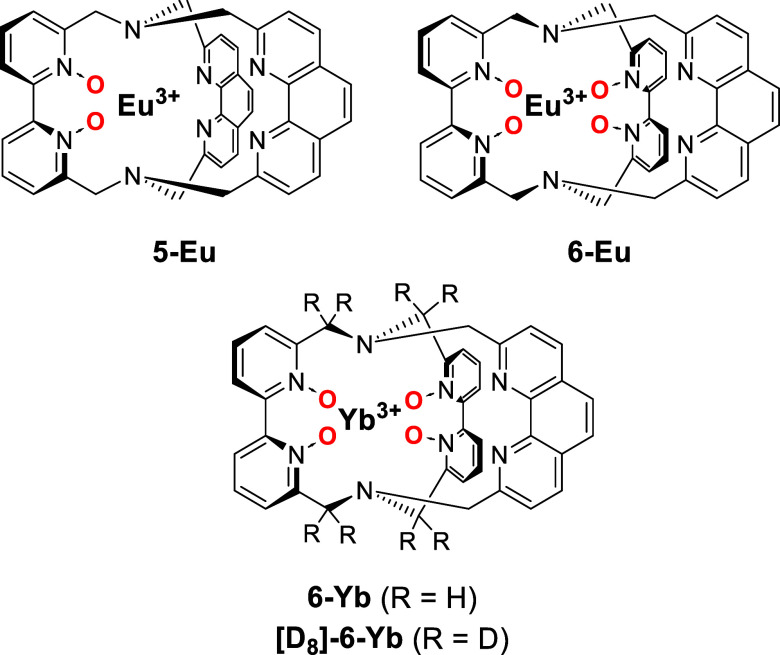
New lanthanoid cryptates **5-Eu**, **6-Eu**, **6-Yb**, and **[D**_**8**_ **]-6-Yb** with different contents of (partially deuterated) pyridine-*N*-oxide moieties and 1,10-phenanthroline units used in this study.

## Results and Discussion

### Cryptate Synthesis

The usual approach for synthesizing macrobicyclic cryptates is the reaction of diamines and dibromides in appropriate ratios (Scheme 1). For the synthetic realization of the desired cryptands **5** and **6**, different building blocks are essential. Benzylic phenanthroline diamine **7** was obtained as trihydrobromide salt by the Delépine reaction of dibromide **8** with hexamethylenetetramine followed by acid hydrolysis of the resulting quaternary ammonium salt.^11^ The known dibromide precursors **8** ^12^ and **9** ^13^/**[D**_**4**_ **]-9** ^14^ were synthesized according to known literature procedures.

**Scheme 1:**
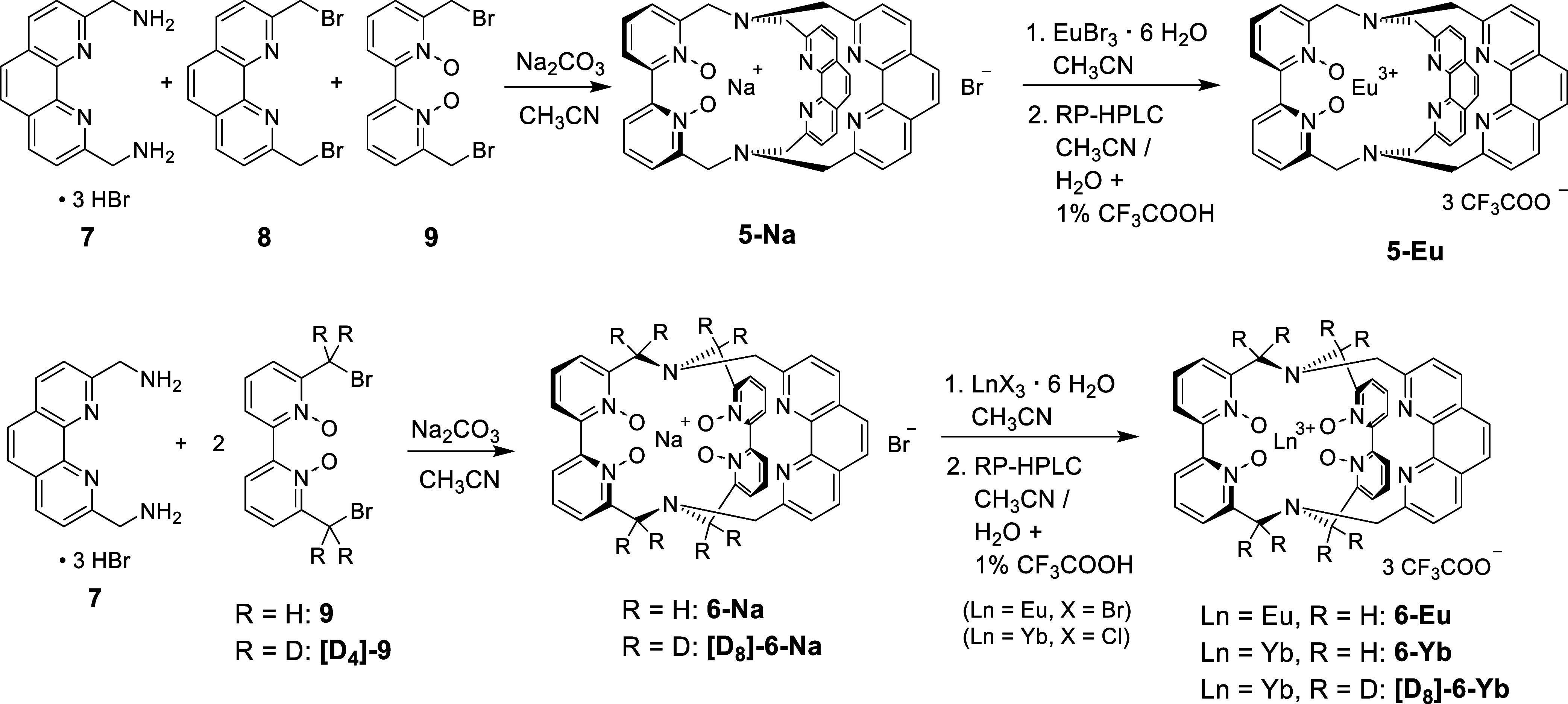
Synthesis of the Lanthanoid Cryptates **5-Eu**, **6-Eu**, **6-Yb**, and **[D**_**8**_ **]-6-Yb**

The sodium cryptate **5-Na** which features two 1,10-phenanthroline motifs, was obtained by reacting the building blocks **7**, **8** and **9** in an equimolar ratio with sodium carbonate in acetonitrile (Scheme 1). This is, to the best of our knowledge, the first reported one-step cryptate synthesis using three different precursors. Unsurprisingly, two other cryptate species, **6-Na** and the sodium cryptate with the known cryptand [phen.phen.phen][Bibr ref15] occurred as byproducts of this one-step macrobicycle formation which could be separated by column chromatography. Nevertheless, the obtained isolated yield for **5-Na** of 26% (see the Experimental Section) is quite respectable for such a complicated reaction and this approach proved the most economical synthetic route to **5-Na**. Macrocyclization using precursors **7** and either **9** or the partially deuterated isotopologue **[D**_**4**_ **]-9** in appropriate ratios (1:2) gave sodium cryptates **6-Na** and **[D**_**8**_ **]-6-Na**, both containing two 2,2′-bipyridine-*N,N*′-dioxide- and one 1,10-phenanthroline unit (Scheme 1).

It is important to purify the sodium complexes **6-Na** via column chromatography on aluminum oxide to avoid Ca^2+^ contamination (present in commerical silica).^16^ All sodium cryptates **5-Na** and **6-Na**/**[D**_**8**_ **]-6-Na** could be converted to the corresponding lanthanoid species by metal exchange with europium tribromide hexahydrate or ytterbium trichloride hexahydrate in boiling acetonitrile. Reversed-phase HPLC purification (see the Supporting Information for more details) of the obtained crude mixtures yielded **5-Eu**, **6-Ln** (Ln = Eu, Yb), and **[D**_**8**_ **]-6-Yb** as trifluoroacetate salts.

It should be noted here that all new lanthanoid cryptates exhibit (in alignment with the well-known **1-Eu** and **2-Eu** ^5^) excellent (kinetic) stability under the relatively harsh HPLC conditions (H_2_O + 1% CF_3_COOH/CH_3_CN). The lanthanoid cryptates are highly soluble in polar, protic solvents (e.g., H_2_O, CH_3_OH), slightly soluble in polar, aprotic solvents (e.g., CH_3_CN) and almost insoluble in nonpolar solvents (e.g., CH_2_Cl_2_). In addition to the europium and ytterbium complexes, the diamagnetic lutetium analogues **5-Lu** and **6-Lu** were obtained as diamagnetic and f–f photoinactive control compounds by the same synthetic procedures (see the Experimental Section).

### Structural Properties

Single crystals suitable for X-ray analysis were obtained by layering a methanolic solution of sodium cryptate **6-Na** with diethyl ether. The cryptate structure shows an eight-coordinate sodium center in an overall *C*_2_-symmetric complex (Figure 4). The sodium cation is completely shielded by the cryptand and does not bind to any additional, external ligand. As expected, the molecular structure exhibits the twisted 2,2′-bipyridine-*N,N*′-dioxide motifs and the rigidly planar 1,10-phenanthroline in the cryptate scaffold.

**Figure 4:**
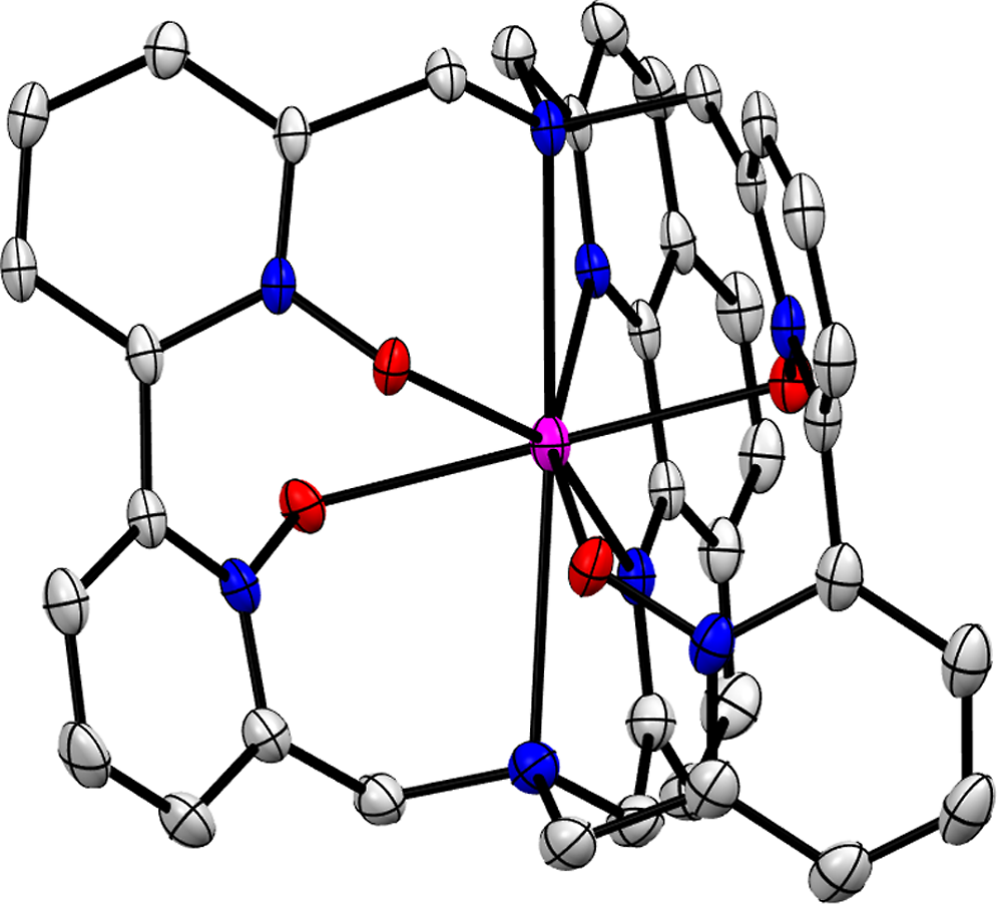
Thermal ellipsoid plot (Ortep 3 for Windows,^17^ 50% probability level) for the cation of **6-Na** (CCDC No. 2184715). The hydrogen atoms, the free bromide anion and the isolated water and methanol molecules are omitted for clarity.

The binding properties of the phenanthroline are unremarkable and resemble the ones found in the crystal structure of the sodium cryptate with the known cryptand [phen.phen.phen].^15b^ Unfortunately, we were not successful in obtaining single crystals suitable for X-ray analysis of **5-Na** and the lanthanoid cryptates.

NMR investigation of the cryptates in solution was possible for all cryptates. ^1^H NMR spectroscopy clearly exhibited one *C*_2_-symmetric cryptate species in CD_3_OD in each case (e.g., see the NMR spectra of **5-Eu** and **6-Eu** in Figure 5). ^1^H NMR spectra of sodium cryptates **5-Na** and **6-Na** showed three well-defined AB systems for the benzylic methylene protons which are consistent with other *N*-oxide containing tris­(biaryl) cryptates with atropisomeric stereogenic elements (see Figures S2 and S3 in the Supporting Information for more details).

**Figure 5:**
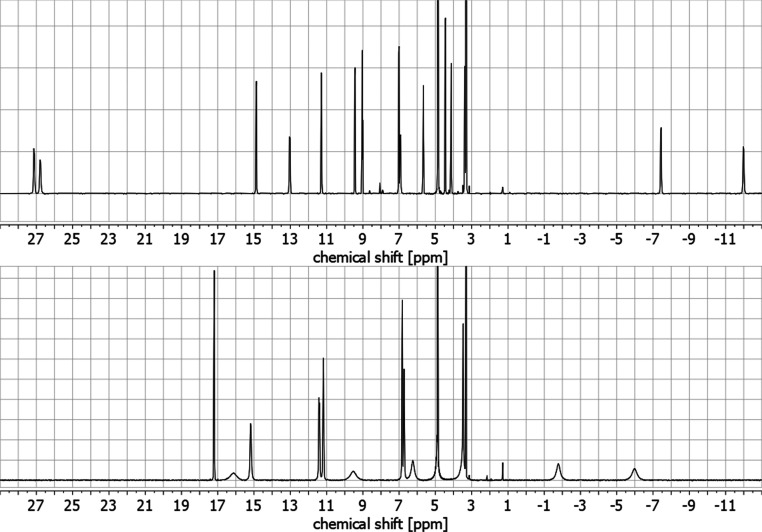
^1^H NMR spectra (CD_3_OD, 400 MHz) of **5-Eu** (top) and **6-Eu** (bottom).

### Photophysical Properties

#### Ligand-Centered Photophysics

To achieve maximum similarity between the present and our previous studies, the europium and ytterbium cryptates were mainly investigated in D_2_O^5^ and in CD_3_OD,^4^ respectively. The UV/vis absorption spectra of **5-Eu** and **6-Ln** (see Figures S15 and S16 in the Supporting Information) show characteristic bands in the spectral region from 280 to ca. 360 nm, even though the bands are slightly different dependent on the phenanthroline content of the current cryptate. The spectrum of **5-Eu**, featuring two phenanthroline motifs, extends a little bit further into the long-wavelength range. To get further insight into the energy transfer from the cryptands to the lanthanoids, we investigated the corresponding lutetium cryptates **5-Lu** and **6**-**Lu** by determining their zero-phonon T_1_ → S_0_ transition energy *E*(T_1_) by low-temperature (77 K) emission spectra (see Figure S17 in the Supporting Information). The lack of vibronic structures of the phosphorescence bands prevents the exact determination of *E*(T_1_). A rough estimate, however, of the possible range for **5-Lu** is *E*(T_1_) ≈ 20,200–21,000 cm^–1^, which is in the range of tris­(bipyridine) cryptand **1** (*E*(T_1_) ≈ 20,400 cm^–1^).^4^ Tetra *N*-oxide cryptate **6-Lu** exhibits a significantly higher triplet level, *E*(T_1_) ≈ 23,500–25,000 cm^–1^ (400–425 nm), which is in agreement with measurements of tris­(bipyridine) cryptates in our previous study where a higher *N*-oxide content afforded a higher triplet energy (cryptand **2** (*E*(T_1_) ≈ 22,600 cm^–1^).^4^ The measured triplet levels in **5-Lu** and **6-Lu** are both well above the emitting ^5^D_0_ state of Eu^3+^ (^5^D_0_ ≈ 17,300 cm^–1^),^18^ and the excited ^2^F_7/2_ level of Yb^3+^ (^2^F_7/2_ ≈ 10,300 cm^–1^),^19^ thus avoiding any thermal energy back transfer from the lanthanoid center to the ligand-centered triplet state.

#### Europium

Steady-state emission of cryptates **5-Eu** and **6-Eu** in D_2_O show strong luminescence with the characteristic europium bands originating from the excited ^5^D_0_ state (Figure 6). Both spectra exhibit only one single peak for each ^5^D_0_ → ^7^F_0_ transition, suggesting the presence of only one emitting species in solution in each case. Along the lines of the tris­(bipyridine) analogues,^5^ an increase in *N*-oxide content is accompanied by an increase in the ^5^D_0_ → ^7^F_0_ energies (see Table 1 and Figure 6). Due to their high sensitivity to the coordination environment, europium spectra contain a lot of information on the molecular structure in solution.^20^ The obtained bands (Figure 6) validate the observed *C*_2_-symmetry by ^1^H NMR spectroscopy in solution (vide supra). The splitting patterns of the emissive transitions indicate a coordination environment with low symmetry around the trivalent europium cation.^20^ In particular, the transition ^5^D_0_ → ^7^F_0_ is comparably quite strong in **5-Eu** and **6-Eu** which could be interesting for coherent spectroscopy and quantum information applications in the future.^21^ Steady-state emission spectra in the solid state (powders, 298 K, λ_exc_ = 532 nm) yield similar spectra compared to the solution data (see Figure S18 in the Supporting Information).

**Figure 6:**
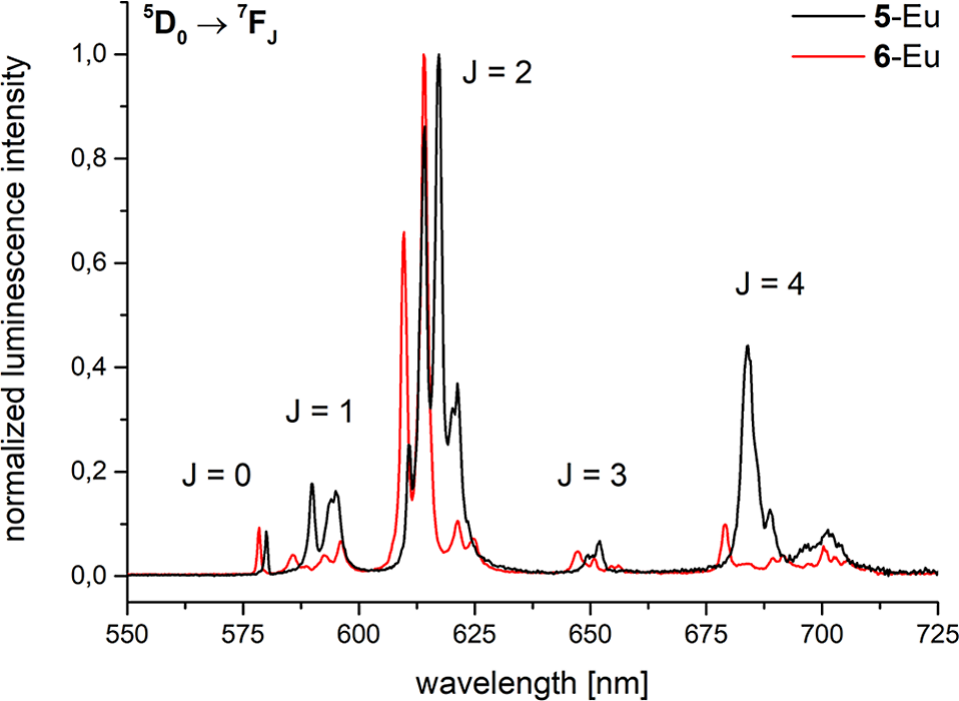
Steady-state emission spectra (D_2_O, *c* ≈ 10 μM) for **5-Eu** (black, λ_exc_ = 300 nm) and **6-Eu** (red, λ_exc_ = 318 nm).

**Table 1:** Luminescence Data for the Europium Cryptates **5-Eu** and **6-Eu** in D_2_O (H_2_O)

compd.	ν̃(D05→F70) [cm^–1^]	τ_obs(D)_ (τ_obs(H)_) [ms]	*q* ^b^	τ_rad_ [ms]	$Q_{Eu}^{Eu}=\frac{\tau_{D}}{\tau_{rad}}$ [%]	*Q*_Eu_^L^ [%]	η_sens_ [%]
**1-Eu** ^5^	17283	1.61 (0.54)	1.2	5.3	30	7.6	25
**2-Eu** ^5^	17301	1.00 (0.43)	1.3	3.6	28	4.2	15
**5-Eu**	17241	1.33 (0.71)^a^	0.5	2.9^c^	46^d^	36 (20)^e^	78^f^
**6-Eu**	17301	1.00 (0.57)^a^	0.6	2.0^c^	50^d^	42 (20)^e^	83^f^

a Luminescence lifetimes: λ_exc_ = 300 nm, λ_em_ = 615 nm (^5^D_0_ → ^7^F_2_), estimated uncertainty ±10%.

b Number of inner-sphere water molecules, see ref 23.

c Radiative lifetime calculated using eq 3, estimated uncertainty ±20%.

d Intrinsic quantum yield, estimated uncertainty ±30%.

e Absolute quantum yield, measured using quinine sulfate (in 0.1 M H_2_SO_4_) as standard (ref 25), values in H_2_O in parentheses, estimated uncertainty ±10%.

f Sensitization efficiency (see eq 1).

The luminescence lifetimes for the europium cryptates were measured in D_2_O (τ_D_2_O_) and H_2_O (τ_H_2_O_) at ambient temperature (see Table 1). For both cryptates, the determined lifetimes in D_2_O (τ_D_2_O_(**5-Eu**) = 1.33 ms; τ_D_2_O_(**6-Eu**) = 1.00 ms) are roughly twice as high as the measured values in H_2_O (τ_H_2_O_(**5-Eu**) = 0.71 ms; τ_H_2_O_(**6-Eu**) = 0.57 ms) which is not surprising due to quenching high-energy O–H stretching vibration overtones in aqueous solution. The observed values for τ in D_2_O and H_2_O as well as the magnitude of the isotope effect in deuterated solvents are very comparable to the ones previously reported for *N*-oxide containing tris­(bipyridine) europium cryptates (see Table 1).^5,22^ Based on the measured lifetimes, the number of inner-sphere water molecules q were evaluated using the empirical formula introduced by Beeby et al.^23^ For **5-Eu** and **6-Eu**, the estimation of q yielded values of 0.5 and 0.6 respectively. The apparent number of inner-sphere water molecules are low and quite different compared to the values obtained for cryptates **1-Eu** (*q* = 1.2) and **2-Eu** (*q* = 1.3).^5^ The only difference between the previously reported bipyridine-based cryptates and the ones investigated here is the presence of 1,10-phenanthroline. The bulkier phenanthroline, compared to bipyridine, may have a positive impact on shielding the metal center from coordinating solvent molecules. It should, however, also be mentioned that the empirical formula commonly used has primarily been developed for europium complexes with aminocarboxylate ligands.^23^ It has been observed in the past that equations of this type for complexes with other ligand classes such as cryptates do not work as well for the unambiguous determination of *q* values.^24^ For the reasons outlined, the obtained, noninteger *q* values or **5-Eu** and **6-Eu**, should not be overinterpreted and do not permit conclusive statements on the inner-sphere compositions at the present time.

Our core interest in this work is to achieve a further reduction of the radiative lifetime τ_rad_ and to receive more knowledge of how τ_rad_ can be utilized for lanthanoid complexes with high luminescence efficiencies. In case of europium­(III), the estimation of τ_rad_ is a lot easier than for other lanthanoid cations because of its emissive transition ^5^D_0_ → ^7^F_1_ which is magnetic-dipole (MD) allowed. If this transition is taken as constant and independent of the ligand environment, the radiative lifetime can easily be determined experimentally by using eq 3, which relates the total integrated emission from the ^5^D_0_ level (*I*_tot_) to the integrated emission of the ^5^D_0_ → ^7^F_1_ band (*I*_MD_).^2b^

3 $$\frac{1}{\tau_{rad}}=A_{MD,0}\cdot n^{3}\cdot \frac{I_{tot}}{I_{MD}}$$

Thus, the radiative lifetime can be estimated from the corrected emission spectrum, the spontaneous emission probability for the ^5^D_0_ → ^7^F_1_ transition in vacuo *A*_MD,0_ = 14.65 s^–1^ (see ref 2b) and the refractive index n. In agreement with the initial hypothesis of our study, phenanthroline does indeed have a positive effect on the radiative lifetime: Compared to the tris­(bipyridine) cryptates **1-Eu** with two pyridine-*N*-oxides (τ_rad_ = 5.3 ms) and **2-Eu** with four pyridine-*N*-oxides (τ_rad_ = 3.6 ms),^5^ the radiative lifetime upon incorporation of phenanthroline moieties into the cryptand scaffolds decreases even further for **5-Eu** with two pyridine-*N*-oxides (τ_rad_ = 2.9 ms) and finally to the lowest value for **6-Eu** with four pyridine-*N*-oxides (τ_rad_ = 2.0 ms). For comparison, we also studied the emission spectrum in the solid state. With the assumption of *n* = 1.5 (see ref 2c) and using eq 3, τ_rad_ can also be obtained. Qualitatively, also very short radiative lifetimes are observed (**5-Eu**: τ_rad_ = 1.6 ms/**6-Eu**: τ_rad_ = 1.8 ms). There is a clear trend from **1-Eu** to **6-Eu** in solution: Higher *N*-oxide- as well as phenanthroline-contents result in shorter radiative lifetimes τ_rad_ and therefore make the emission more competitive compared to nonradiative deactivation processes. Combining the two effects reduces τ_rad_ by an impressive 62% going from **1-Eu** to **6-Eu**.

Using the measured lifetimes τ_obs_ in D_2_O (see text above or Table 1) and the radiative lifetimes obtained above, the intrinsic quantum yields in D_2_O according to eq 2 amount to *Q*_Ln_^Ln^ = 46% for **5-Eu** and to *Q*_Ln_^Ln^ = 50% **6-Eu** (Table 1) which is a significant improvement compared to **1-Eu** (*Q*_Ln_^Ln^ = 30%) and **2-Eu** (*Q*_Ln_^Ln^ = 28%)^5^ and illustrates the potential of the rigidly planar phenanthroline units in both new europium cryptates. Concomitantly, there is a second benefit of introducing phenanthroline into the cryptand scaffolds: As can be seen from Table 1, the negative impact of increasing the *N*-oxide content on the already low sensitization efficiencies η_sens_ seen in tris­(bipyridine)-based cryptates **1-Eu** (η_sens_ = 25%) and **2-Eu** (η_sens_ = 15%) is absent. Instead, the phenanthroline-based cryptates show a much higher sensitization efficiency η_sens_ for **5-Eu** (η_sens_ = 78%) and η_sens_ does not drop with an increase in *N*-oxide content going to **6-Eu** (η_sens_ = 83%). The absolute quantum yields *Q*_Eu_^L^ of the phenanthroline-containing cryptates in D_2_O were measured against quinine sulfate as standard.^25^ The quantum yields are very high (Table 1: *Q*_Eu_^L^ = 36% for **5-Eu**; *Q*_Eu_^L^ = 42% for **6-Eu**) and stay very good even in H_2_O solution (Table 1: *Q*_Eu_^L^ = 20% for both **5-Eu** and **6-Eu**). With cryptates of this type already being known to be suitable for biomedical applications,^26^ this particular result bodes well for future applicability of the new europium luminophores. In fact, the obtained absolute quantum yield are among the highest values for europium luminophores in aqueous solution.^27^ It is quite striking that a simple substitution of 1,10-phenanthroline for 2,2′-bipyridine is responsible for the up to 10-fold increase of absolute quantum yield (Table 1: e.g. absolute quantum yield increase in D_2_O from *Q*_Eu_^L^ = 4.2% for **2-Eu** to *Q*_Eu_^L^ = 40% for **6-Eu**). There are several photophysical benefits that synergistically combine when replacing phenanthroline units for some of the bipyridine moieties in these cryptates. Based on the results, we can confirm that phenanthroline substantially lowers the radiative lifetime τ_rad_ for Eu^3+^ compared to the analogous cryptates featuring only bipyridine-based units which will prove a useful design principle for future improvements in luminophores of this type. The biggest impact, however, seems to be the considerable increase in η_sens_ for the new europium cryptates with mixed phenanthroline/bipyridine antennae. It appears plausible that phenanthroline with its rigid, planar framework^9^ allows less nonradiative deactivation before the energy transfer step from the ligand to the lanthanoid-centered excited states but this alone seems unlikely to account for the massive increase in sensitization efficiency. Especially the energies of critical states (e.g., potential CT states) which are involved within the sensitization pathway could be responsible for the observed enhancement. The lack of current understanding will certainly merit more in-depth investigations in the future.

#### Ytterbium

The ytterbium cryptates **6-Yb** and **[D**_**8**_ **]-6-Yb** expectedly show almost identical, normalized near-IR luminescence bands for the transition ^2^F_5/2_ → ^2^F_7/2_ (λ_em_ ≈ 1000 nm) in CD_3_OD after ligand excitation in the UV region (Figure 7).

**Figure 7:**
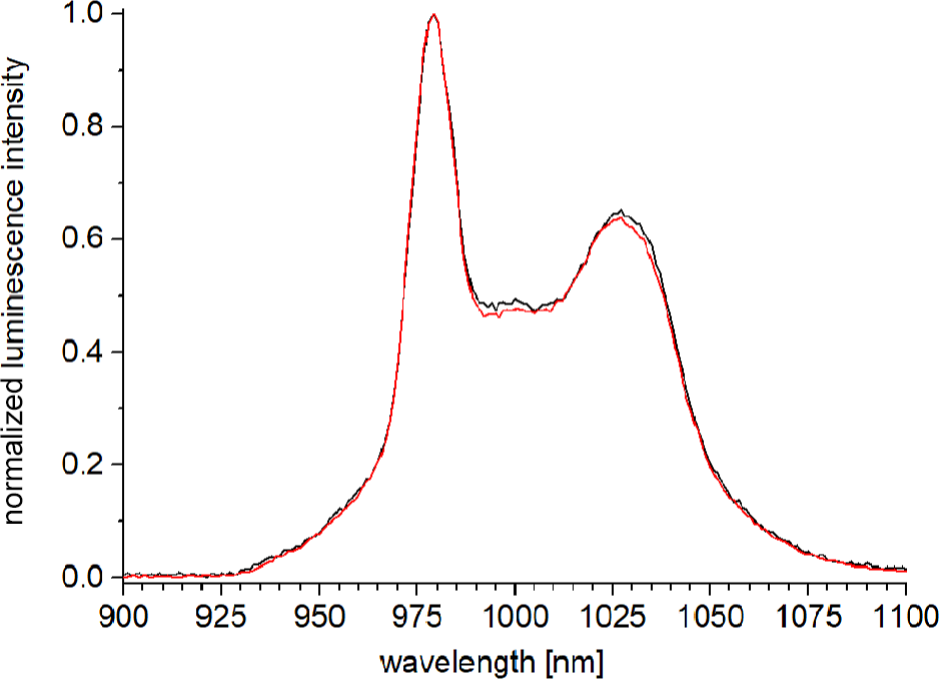
Normalized steady-state emission spectra (CD_3_OD, λ_exc_ = 305 nm, *c* ≈ 10 μM) for **6-Yb** (black) and **[D**_**8**_ **]-6-Yb** (red).

Luminescence lifetime measurements (see Table 2) under the same conditions revealed the influence of the reduced multiphonon quenching in **[D**_**8**_ **]-6-Yb** (τ_obs_ = 34 μs) compared to **6-Yb** (τ_obs_ = 25 μs). This observation is also manifestly visible in the increase in absolute quantum yields (Table 2: *Q*_Yb_^L^ = 2.2% for **6-Yb**; *Q*_Yb_^L^ = 2.7% for **[D**_**8**_ **]-6-Yb**). These quantum efficiencies are very respectable compared to other, known ytterbium luminophores in the literature.^1,2^ In order to get a more detailed picture of the photophysical parameters, the radiative lifetimes were also determined in CD_3_OD.

**Table 2:** Luminescence Data for Ytterbium Cryptates **6-Yb** and **[D**_**8**_ **]-6-Yb** in CD_3_OD

compound	τ_obs_ [μs]	τ_rad_ [μs]	*Q*_Yb_^Yb^ [%]	*Q*_Yb_^L^ [%]	η_sens_ [%]
**1-Yb** ^4^	12	1250	-	-	68
**2-Yb** ^4^	26	658	-	-	46
**6-Yb**	25^a^	612^b^	4.1^c^	2.2^d^	52^e^
**[D**_**8**_ **]-6-Yb**	34^a^	612^b^	5.5^c^	2.7^d^	49^e^

a Luminescence lifetimes: λ_exc_ = 305 nm, λ_em_ = 979 nm (^2^F_5/2_ → ^2^F_7/2_), estimated uncertainty ±10%.

b Radiative lifetime calculated using the equation in the Supporting Information (see Section 5), estimated uncertainty ±20%.

c Intrinsic quantum yield, estimated uncertainty ±30%.

d Absolute quantum yield, measured using [Yb­(TTA)_3_phen] in toluene as standard (ref 28), estimated uncertainty ±20%.

e Sensitization efficiency (see eq 1).

In contrast to Eu^3+^, where the special properties of the MD transition ^5^D_0_ → ^7^F_1_ could be used to conveniently calculate τ_rad_ from the emission spectrum directly^2b^ (vide supra), in the case of Yb^3+^ τ_rad_ for ^2^F_5/2_ → ^2^F_7/2_ was derived from the quantitative evaluation of the absorption band ^2^F_7/2_ → ^2^F_5/2_.^2b^ The corresponding spectra for both isotopologues **6-Yb** and **[D**_**8**_ **]-6-Yb** are almost identical and show molar extinction coefficients ε < 10 M^–1^ cm^–1^ which are typical for the Laporte-forbidden but spin-allowed transition in question (Figure 8). A comparison of the f–f absorption and emission spectra for **6-Yb** shows the complementary fine-structure expected (Figure 9). Using a modified Einstein equation^2b^ allowed the calculation of τ_rad_ (see the Supporting Information for details) for the new cryptates. **6-Yb** and **[D**_**8**_ **]-6-Yb** expectedly show the same τ_rad_ = 612 μs (Table 2). The obtained value is very short for molecular ytterbium complexes in solution.^1,2^ The most relevant comparison can be made between **6-Yb** to the already very successul luminophore **2-Yb** ^4^ which in its perdeuterated form had an impressive quantum yield *Q*_Yb_^L^ = 12%.^4^ Both systems seem very similar in a number of important photophysical parameters, e.g. τ_obs_ = 26 μs/τ_rad_ = 612 μs/η_sens_ = 52% for **6-Yb** vs τ_obs_ = 25 μs/τ_rad_ = 658 μs and η_sens_ = 46% for **2-Yb** (Table 2). In essence, the introduction of the phenanthroline motiv is not as effective for the improvement of ytterbium luminescence as it was shown for the corresponding europium cryptates **2-Eu** and **6-Eu** (vide supra), in part because **2-Yb** (unlike **2-Eu**) already showed high emission efficiency.

**Figure 8:**
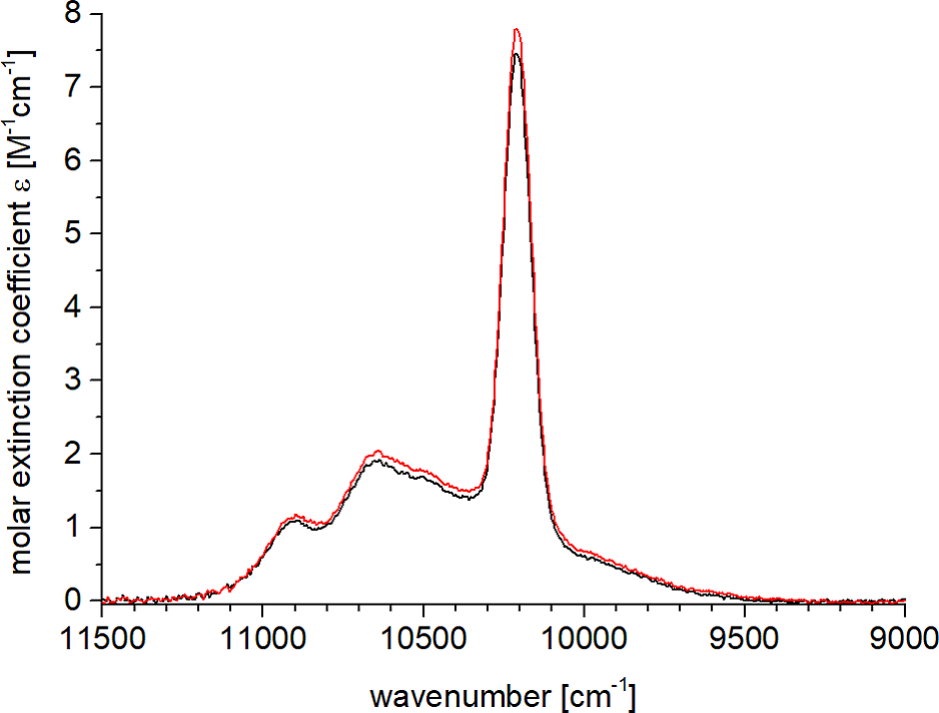
Absorption spectra (CD_3_OD, *c* ≈ 3 mM) for the f–f transition ^2^F_7/2_ → ^2^F_5/2_ in **6-Yb** (black) and in **[D**_**8**_ **]-6-Yb** (red).

**Figure 9:**
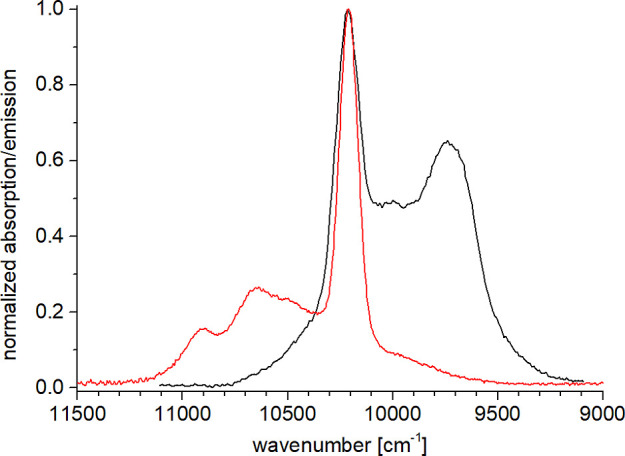
Comparison of the normalized absorption (red) and emission spectra (black, λ_exc_ = 305 nm) for the f–f transition ^2^F_7/2_ ↔ ^2^F_5/2_ of **6-Yb** in CD_3_OD.

## Conclusion

In conclusion, we were able to integrate the rigidly planar 1,10-phenanthroline moiety into tris­(biaryl)-*N,N*′-dioxide cryptand scaffolds. We have synthesized two europium­(III) cryptates with different amounts of 1,10-phenanthroline- and 2,2′-bipyridine-*N,N*′-dioxide building blocks in their macrobicyclic frameworks, as well as two isotopologic, partially deuterated ytterbium­(III) cryptates of one of the new phenanthroline cryptates. The Yb cryptates show very short radiative lifetimes τ_rad_, moderate sensitization efficiencies η_sens_ and good absolute quantum yields *Q*_Yb_^L^ but they do not significantly outperform their all-bipyridine counterparts. In contrast, replacing 2,2′-bipyridine by 1,10-phenanthroline in the europium cryptates leads to a considerable reduction of τ_rad_ but also a remarkable improvement in η_sens_ and *Q*_Eu_^L^. Absolute quantum yields of up to 42% in D_2_O and 20% in H_2_O for **6-Eu** show great promise for the large number of lanthanoid luminescence applications in aqueous media, for example biomolecule labeling or time-resolved fluorescence energy transfer.

## Experimental Section

### General Information

Unless stated otherwise, starting materials and reagents were purchased from commercial suppliers and used as received. Airsensitive reactions were carried out under an inert atmosphere of argon using Schlenk technique. All solvents used for synthesis and purification were of HPLC grade purity. CH_3_CN was dried using a MBraun SPS-800 solvent purification system. Deuterated solvents had deuterium contents >99.5%D. Lanthanoid salts with 99.99% purity (REO) were used for the preparation of europium and lutetium cryptates, for ytterbium the purity was 99.995% (REO). Column chromatography was performed using silica gel 60 (Merck, 40–63 μm), aluminum oxide 60 (Acros Organics, neutral, Brockmann I, 40–300 μm) or aluminum oxide 90 (Macherey-Nagel and Merck, neutral and basic, Brockmann I, 63–200 μm). Analytical thin layer chromatography (TLC) was done on silica gel 60 F_254_ plates (Merck, coated on aluminum sheets) or aluminum oxide 60 F_254_, neutral plates (Supelco, coated on aluminum sheets).

NMR spectra were recorded on Bruker Avance III HD 300 (^1^H: 300 MHz), Bruker AVII+400 (^1^H: 400 MHz), Bruker AVII+500 (^1^H: 500 MHz), Bruker Avance III HDX 600 (^1^H: 600 MHz) or Bruker Avance III HDX 700 (^1^H: 700 MHz) spectrometers. All chemical shifts (δ) are reported in parts per million (ppm) relative to tetramethyl silane (^1^H, ^13^C) and trichlorofluoromethane (^19^F). The solvents’ residual signals were used as internal reference (^1^H, ^13^C). Observed signal multiplicities and broad lines are specified as s (singlet), d (doublet), t (triplet), q (quartet), m (multiplet), br (broad).

ESI mass spectra were measured by the Analytics Department of the University of Tübingen using Bruker amaZon SL and Bruker maXis.

Elemental analysis was performed by the Central Analytical Facility of the Chemistry Department of the University of Tübingen with a VarioMicro V1.9.2. analysis system.

Reversed-phase­(RP)-HPLC was performed using Lichrospher RP-18e columns (Merck, semipreparative: 250 mm × 10 mm, 10 μm particle size; analytical: 125 mm × 4 mm, 5 μm particle size) on a Knauer AZURA P6.1L system. For more details see the Supporting Information.

### Synthesis

#### Diamine **7**

A solution of hexamethylenetetramine (575 mg, 4.1 mmol, 5.0 equivs) in CHCl_3_ (40 mL, HPLC grade) was refluxed and 2,9-bis­(bromomethyl)-1,10-phenanthroline (**8**)^12^ (300 mg, 0.82 mmol, 1.0 equiv) in CHCl_3_ (60 mL, HPLC grade) was added dropwise. A colorless precipitate formed and the mixture was refluxed for additional 2 h. The mixture was allowed to cool and to stand for 16 h at room temperature. The precipitate was removed by filtration, washed with CHCl_3_ (25 mL, HPLC grade) and dried in vacuo. Addition of a mixture of water (2.1 mL), ethanol (8.3 mL) and 48% HBr (2.5 mL) at 82 °C (bath temperature) yielded a bronze colored solution. The solution was allowed to cool down overnight and the bronze colored, crystalline platelets were filtered off, washed with cold ethanol and dried (89 mg, 0.18 mmol, 22%).

^1^H NMR (400 MHz, [D_6_]-DMSO): δ 8.63 (d, *J* = 8.3 Hz, 2H), 8.51 (s br, 6H), 8.09 (s, 2H), 7.90 (d, *J* = 8.2 Hz, 2H), 4.58 (q, *J* = 5.8 Hz, 4H) ppm. ^13^C NMR (63 MHz, DMSO-*d*_6_): δ 153.4, 143.5, 137.7, 128.1, 126.7, 122.1, 43.1 ppm. MS (ESI, pos. mode): *m*/*z* (%) = 239.2 (100, [M + H]^+^). Elemental analysis: Anal. Calcd for C_14_H_17_N_4_Br_3_ (*M*_r_ = 481.03): C, 34.96; H, 3.56; N, 11.65. Found: C, 34.69; H, 3.53; N, 11.30.

#### Sodium Cryptate **5-Na**

A suspension of dibromide **8** ^12^ (30 mg, 82 μmol, 1.0 equiv), diamine **7** (40 mg, 83 μmol, 1.0 equiv), dibromide **9** ^13^ (31 mg, 82 μmol, 1.0 equiv) and Na_2_CO_3_ (119 mg, 1.12 mmol, 13.7 equivs) in CH_3_CN (300 mL, HPLC grade) was heated under reflux for 42 h. The heating bath was removed, the hot solution was filtered and all volatiles of the filtrate were removed in vacuo. The resulting residue was subjected to column chromatography (SiO_2_, CH_2_Cl_2_/CH_3_OH, gradient 9:1 → 7:1) yielding the title compound as a colorless solid (15 mg, 21 μmol, 26%).

^1^H NMR (400 MHz, CD_3_OD): δ 8.41 (d, *J* = 8.2 Hz, 2H), 8.38 (d, *J* = 8.2 Hz, 2H), 7.93 (dd, *J* = 7.9, 2.0 Hz, 2H), 7.88 (dd, *J* = 10.3, 8.8 Hz, 4H), 7.83 (dd, *J* = 7.8, 2.0 Hz, 2H), 7.73 (d, *J* = 5.2 Hz, 2H), 7.71 (d, *J* = 5.1 Hz, 2H), 7.65 (t, *J* = 7.8 Hz, 2H), 4.56 (d, *J* = 11.7 Hz, 2H), 4.40 (d, *J* = 14.8 Hz, 2H), 4.09 (d, *J* = 14.7 Hz, 2H), 3.98 (d, *J* = 14.8 Hz, 2H), 3.87 (d, *J* = 15.0 Hz, 2H), 3.58 (d, *J* = 11.6 Hz, 2H) ppm. ^13^C NMR (100 MHz, CD_3_OD): δ 160.2, 159.4, 150.5, 147.3, 147.0, 146.5, 138.7, 138.7, 131.4, 129.8, 129.8, 128.6, 128.5, 127.4, 127.4, 124.8, 124.7, 63.0, 61.5, 55.7 ppm. MS (ESI pos. mode): *m*/*z* (%) = 677.5 (100, [M]+). *R*_f_ = 0.5 (SiO_2_, CH_2_Cl_2_/MeOH 4:1, UV detection).

#### Sodium Cryptate **6-Na**

A suspension of diamine **7** (60 mg, 125 μmol, 1.0 equiv), dibromide **9** ^13^ (94 mg, 250 μmol, 2.0 equivs) and Na_2_CO_3_ (133 mg, 1.25 mmol, 10.0 equivs) in CH_3_CN (250 mL, HPLC grade) was heated under reflux for 40 h. The heating bath was removed, the hot solution was filtered and all volatiles of the filtrate were removed in vacuo. Column chromatography (neutral alumina 90, activity I, CH_2_Cl_2_/CH_3_OH 20:1) yielded the cryptate as yellowish, iridescent platelets (60 mg, 78 μmol, 63%).

Single crystal suitable for X-ray diffraction were grown from methanolic solution of **6-Na** by slow vapor diffusion of Et_2_O.

^1^H NMR (400 MHz, CD_3_OD): δ 8.39 (d, *J* = 8.4 Hz, 2H), 7.91 (dd, *J* = 7.7, 2.1 Hz, 2H), 7.87 (s, 2H), 7.80 (dd, *J* = 8.0, 1.9 Hz, 2H), 7.75–7.64 (m, 8H), 7.49 (t, *J* = 7.9 Hz, 2H), 4.59 (d, *J* = 12.0 Hz, 2H), 4.42 (d, *J* = 11.6 Hz, 2H), 4.14 (d, *J* = 15.2 Hz, 2H), 4.00 (d, *J* = 15.6 Hz, 2H), 3.53 (d, *J* = 12.0 Hz, 2H), 3.53 (d, *J* = 12.0 Hz, 2H) ppm. ^13^C NMR (100 MHz, CD_3_OD): δ 159.6, 149.6, 149.5, 147.3, 146.9, 146.2, 138.9, 131.4, 130.7, 130.3, 129.2, 128.9, 127.8, 127.7, 126.9, 124.9, 63.1, 56.6, 55.8 ppm. MS (ESI, pos. mode): *m*/*z* (%) = 685.3 (100, [M]^+^). *R*_f_ = 0.2 (neutral Al_2_O_3_, CH_2_Cl_2_/CH_3_OH 20:1, UV detection).

#### Sodium Cryptate **[D**_**8**_ **]-6-Na**

A suspension of diamine **7** (50 mg, 0.10 mmol, 1.0 equiv), partially deuterated dibromide **[D**_**4**_ **]-9** ^14^ (79 mg, 0.21 mmol, 2.1 equivs) and Na_2_CO_3_ (111 mg, 1.0 mmol, 10.0 equivs) in CH_3_CN (250 mL, HPLC grade) was heated under reflux for 46 h. The heating bath was removed, the hot solution was filtered and all volatiles of the filtrate were removed in vacuo. Column chromatography (neutral alumina 90, activity I, gradient CH_2_Cl_2_/CH_3_OH 50:1 to 20:1) yielded the cryptate as yellowish, iridescent platelets (50 mg, 71 μmol, 71%, 98%D from ESI-MS).

^1^H NMR (400 MHz, CD_3_OD): δ 8.38 (d, *J* = 8.3 Hz, 2H), 7.91 (dd, *J* = 7.7, 2.1 Hz, 2H), 7.86 (s, 2H), 7.80 (dd, *J* = 7.8, 2.0 Hz, 2H), 7.75–7.64 (m, 8H), 7.49 (t, *J* = 7.9 Hz, 2H), 4.14 (d, *J* = 15.2 Hz, 2H), 4.00 (d, *J* = 15.4 Hz, 2H) ppm. MS (ESI, pos. mode): *m*/*z* (%) = 693.3 (100, [M]^+^). *R*_f_ = 0.09 (neutral Al_2_O_3_, CH_2_Cl_2_/CH_3_OH 50:1, UV detection).

#### Europium Cryptate **5-Eu**

Sodium cryptate **5-Na** (12 mg, 16 μmol, 1.0 equiv) and EuCl_3_·6H_2_O (13 mg, 35 μmol, 2.2 equivs) were suspended in CH_3_CN (15 mL, HPLC grade). After stirring the mixture, a colorless precipitate appeared immediately. The suspension was heated under reflux for 45 h, cooled and filtered through a membrane filter (nylon, 0.45 μm pore size). The colorless precipitate was washed with CH_3_CN and dried in vacuo. The crude product was dissolved in a minimum of water, filtered through a membrane filter (nylon, 0.45 μm pore size) and the filtrate was subjected to preparative reversed-phase HPLC (see the Supporting Information for details). Fractions containing pure lanthanoid complexes were combined (retention time *t*_r_ = 21.5 min) and evaporated to dryness at room temperature to yield **5-Eu** as a colorless solid (11 mg, 9.6 μmol, 60%).

^1^H NMR (400 MHz, CD_3_OD): δ 27.12 (d, *J* = 15.68 Hz, 2H), 26.79 (d, *J* = 12.72 Hz, 2H), 14.88 (d, *J* = 8.48 Hz, 2H), 13.03 (d, *J* = 15.26 Hz, 2H), 11.28 (d, *J* = 8.48 Hz, 2H), 9.45–9.43 (m, 2H), 9.03 (t, *J* = 7.84 Hz, 2H), 7.01 (d, *J* = 8.48 Hz, 2H), 6.93 (d br, *J* = 11.44 Hz, 2H), 5.67 (d, *J* = 7.63 Hz, 2H), 4.45 (d, *J* = 8.48 Hz, 2H), 4.13 (d, *J* = 8.05 Hz, 2H), 3.38 (d, *J* = 8.05 Hz, 2H), −7.44 (d, *J* = 12.72 Hz, 2H), −12.00 (d, *J* = 13.99 Hz, 2H) ppm. ^19^F­{^1^H} NMR (376 MHz, CD_3_OD): δ −77.98 (s br) ppm. MS (ESI, pos. mode, addition of HCOOH): *m*/*z* (%) = 403.06 (5, [M + e^–^]^2+^), 426.05 (100, [M + HCOO^–^]^2+^).

#### Europium Cryptate **6-Eu**

Sodium cryptate **6-Na** (30 mg, 39 μmol, 1.0 equiv) and EuCl_3_·6H_2_O (26 mg, 70 μmol, 1.8 equivs) were suspended in CH_3_CN (40 mL, HPLC grade). The slightly yellow suspension was heated under reflux for 46 h. The solvent was removed in vacuo and the crude product was dissolved in a minimum of water, filtered through a membrane filter (nylon, 0.45 μm pore size) and the filtrate was subjected to preparative reversed-phase HPLC (see the Supporting Information for details). Fractions containing pure lanthanoid complexes were combined (retention time t_r_ = 16.9 min) and evaporated to dryness at room temperature to yield **6-Eu** as a colorless solid (11 mg, 9.5 μmol, 24%).

^1^H NMR (400 MHz, CD_3_OD): δ 17.20 (d, 2H), 16.14 (s br, 2H), 15.19 (d, 2H), 11.41 (d, 2H), 11.18 (t, 2H), 9.54 (s br, 2H), 6.83 (d, 2H), 6.73 (m, 2H), 6.24 (s br, 2H), 4.87 (s br, 4H), 3.47 (s br, 4H), −1.78 (s br, 2H), −5.97 (s br, 2H) ppm. ^19^F­{^1^H} NMR (376 MHz, CD_3_OD): δ −76.98 (s) ppm. MS (ESI, pos. mode, addition of HCOOH): *m*/*z* (%) = 407.05 (5, [M + e^–^]^2+^), 430.04 (100, [M + HCOO^–^]^2+^), 452.04 (60), 464.01 (52).

#### Ytterbium Cryptate **6-Yb**

Sodium cryptate **6-Na** (31 mg, 41 μmol, 1.0 equiv) and YbCl_3_·6H_2_O (28.5 mg, 73 μmol, 1.8 equivs) were suspended in CH_3_CN (60 mL, HPLC grade). The slightly yellow suspension was heated under reflux for 68 h. Additional YbCl_3_·6H_2_O (8.0 mg, 21 μmol, 0.5 equivs) was added and heating was continued under reflux for further 14 h. The solvent was removed in vacuo. The crude product was dissolved in a minimum of MeOH and layered with Et_2_O. The precipitate was collected and subjected to preparative reversed-phase HPLC (see the Supporting Information for details). Fractions containing pure lanthanoid complexes were combined (retention time *t*_r_ = 9.1 min) and evaporated to dryness at room temperature to yield **6-Yb** as a colorless solid (12.5 mg, 11 μmol, 27%).

^1^H NMR (400 MHz, CD_3_OD): δ 99.4, 82.4, 72.9, 44.2, 29.7, 14.8, −1.43, −2.76, −7.08, −10.5 ppm. ^19^F­{^1^H} NMR (376 MHz, CD_3_OD): δ −76.86 (s) ppm. MS (ESI, pos. mode, addition of HCOOH): *m*/*z* (%) = 440.58 (100, [M + HCOO^–^]^2+^).

#### Ytterbium Cryptate **[D**_**8**_ **]-6-Yb**

Sodium cryptate **[D**_**8**_ **]-6-Na** (35 mg, 45 μmol, 1.0 equiv) and YbCl_3_·6H_2_O (31.9 mg, 81 μmol, 1.8 equivs) were suspended in CH_3_CN (60 mL, HPLC grade). The slightly yellow suspension was heated under reflux for 50 h. Additional YbCl_3_·6H_2_O (9.0 mg, 23 μmol, 0.5 equivs) was added and heating was continued under reflux for further 50 h. The solvent was removed in vacuo. The crude product was subjected to preparative reversed-phase HPLC (see the Supporting Information for details). Fractions containing pure lanthanoid complexes were combined (retention time t_r_ = 8.8 min) and evaporated to dryness at room temperature to yield the title compound as a colorless solid (17.9 mg, 15 μmol, 33%).

^1^H NMR (400 MHz, CD_3_OD): δ 100.4, 82.0, 44.2, 14.8, −1.33, −2.76, −7.03, ppm. ^19^F­{^1^H} NMR (376 MHz, CD_3_OD): δ −76.84 (s) ppm. MS (ESI, pos. mode, addition of HCOOH): *m*/*z* (%) = 444.60 (100, [M + HCOO^–^]^2+^).

#### Lutetium Cryptate **5-Lu**

Sodium cryptate **5-Na** (5.0 mg, 7.0 μmol, 1.0 equiv) and LuCl_3_·6H_2_O (5.0 mg, 14 μmol, 2.0 equivs) were suspended in CH_3_CN (7 mL, HPLC grade). The mixture was heated under reflux for 45 h. The solvent was removed in vacuo and the crude product was dissolved in a minimum of water, filtered through a membrane filter (nylon, 0.45 μm pore size) and the filtrate was subjected to preparative reversed-phase HPLC (see the Supporting Information for details). Fractions containing pure lanthanoid complexes were combined (retention time *t*_r_ = 16.7 min) and evaporated to dryness at room temperature to yield **5-Lu** as a colorless solid (5 mg, 4 μmol, 56%).

^1^H NMR (400 MHz, CD_3_OD): δ 8.83 (d, *J* = 8.35 Hz, 2H), 8.74 (d, *J* = 8.32 Hz, 2H), 8.38 (dd, *J* = 7.29, 2.34 Hz, 2H), 8.28 (dd, *J* = 7.88, 2.56 Hz, 2H), 8.26–8.23 (m, 2H), 8.15 (dd, *J* = 9.89, 8.89 Hz, 4H), 8.06 (d, *J* = 8.37 Hz, 2H), 7.99 (d, *J* = 8.30 Hz, 2H), 4.97 (d, *J* = 15.33 Hz, 2H), 4.69 (d, *J* = 12.92 Hz, 2H), 4.43 (d, *J* = 15.97 Hz, 2H), 4.35 (d, *J* = 15.46 Hz, 2H), 4.15 (d, *J* = 15.80 Hz, 2H), 4.06 (d, *J* = 12.93 Hz, 2H) ppm.

#### Lutetium Cryptate **6-Lu**

Sodium cryptate **6-Na** (5 mg, 7 μmol, 1.0 equiv) and LuCl_3_·6H_2_O (5 mg, 14 μmol, 2.0 equivs) were suspended in CH_3_CN (7 mL, HPLC grade). The mixture was heated under reflux for 45 h. The solvent was removed in vacuo and the crude product was dissolved in a minimum of water, filtered through a membrane filter (nylon, 0.45 μm pore size) and the filtrate was subjected to preparative reversed-phase HPLC (see the Supporting Information for details). Fractions containing pure lanthanoid complexes were combined (retention times *t*_r_ = 15.0 min) and evaporated to dryness at room temperature to yield the **6-Lu** as a colorless solid (2 mg, 1 μmol, 21%).

^1^H NMR (400 MHz, CD_3_OD): δ 8.67 (d, 2H), 8.16 (dd, *J* = 7.58, 2.40 Hz, 2H), 8.13 (dd, *J* = 7.82, 1.82 Hz, 2H), 8.08–8.05 (m, 4H), 8.03 (dd, *J* = 7.85, 2.41 Hz, 2H), 8.00–7.92 (m, 6H), 4.65 (d, *J* = 12.67 Hz, 2H), 4.38 (d, *J* = 12.17 Hz, 2H), 4.33 (d, *J* = 16.36 Hz, 2H), 4.26 (d, *J* = 16.25 Hz, 2H), 3.89 (d, *J* = 12.10 Hz, 2H), 3.84 (d, *J* = 12.75 Hz, 2H) ppm.

### Photophysical Measurements

UV/vis spectra were measured on a Jasco V-770 spectrophotometer using quartz cuvettes (Suprasil, 1.0 cm path length) at room temperature.

A Horiba Fluorolog-3 spectrofluorometer equipped with a 450 W xenon lamp was used for steady-state measurements. Emission was detected in 90° geometry by a Hamamatsu R2658P PMT detector (200 nm < λ_em_ < 1010 nm) in the visible region or by a Hamamatsu H10330–75 PMT detector (950 nm < λ_em_ < 1700 nm) in the near-IR. Spectral selection in the excitation path was accomplished by a DFX monochromator (double gratings: 1200 grooves/mm, 330 nm blaze) and in the emission paths in the visible/near-IR spectral region (λ_em_ < 1010 nm) by a spectrograph iHR550 (single gratings: either 1200 grooves/mm, 500 nm blaze or 950 grooves/mm, 900 nm blaze) and in the near-IR spectral region (λ_em_ > 950 nm) by a spectrograph iHR320 (single grating: 600 grooves/mm, 1000 nm blaze). Spectral correction of the emission spectra was performed with a correction curve implemented by the instrument manufacturer. Absolute quantum yields *Q*_Ln_^L^ were determined in repeated experiments by the optically dilute method using the following eq 4

4 $$Q_{\mathrm{Ln}}^{L}=\Phi_{r}\cdot \left({Grad}_{x}/{Grad}_{r}\right)\cdot \left(n_{x}^{2}/n_{r}^{2}\right)$$

where *n* is the refractive index (H_2_O = 1.334; D_2_O = 1.328; toluene: *n* = 1.496; CD_3_OD: *n* = 1.326) and Grad is the linearly fitted slope from the plot of the integrated luminescence intensity versus the absorbance at the excitation wavelength. The subscripts x and r refer to the sample and reference, respectively. Quinine sulfate in 0.1 M sulfuric acid was used as reference for Eu emission with a fluorescence quantum yield of Φ_r_ = 54.6%.^25^ For near-IR luminescence, [Yb­(TTA)_3_phen] in toluene was used with a quantum yield of Φ_r_ = 1.1%.^28^

The luminescence decay kinetics were determined at 298 K with a 70 W pulsed xenon flash lamp (pulse width ca. 2 μs fwhm). The analysis of the luminescence decay kinetics (deconvolution, statistical parameters, etc.) was performed using the software package DAS from Horiba.

Low temperature steady state emission spectra were recorded in a glassy matrix of CH_3_OH/EtOH (1:1, v/v) in standard NMR tubes at 77 K (liquid nitrogen) using a low-temperature optical dewar.

Steady-state emission spectra of powder samples of **5-Eu** and **6-Eu** were measured at room temperature and an excitation wavelength of 532 nm (Cobolt Samba 0532–04–01–0100–500) using an Andor Shamrock 500i spectrograph (150 grooves/mm, 800 nm blaze), equipped with an Andor iVac 316B LDC-DD CCD sensor.

## Supplementary Material



## Notes

^1^H NMR spectra, HPLC traces for the lanthanoid complexes, extended photophysical data, crystallographic details for **6-Na**, details for the calculation of τ_rad_ in **6-Yb** and **[D**_**8**_ **]-6-Yb** (PDF)
