# Supplementary material for: Mixed Phenanthroline–Bipyridine Europium and Ytterbium Cryptates—Bright Luminophores with Small Radiative Lifetimes
Source: Inorg Chem. 2026 Feb 22;65(9):5109–18. doi: 10.1021/acs.inorgchem.5c05828 (PMC12977058; doi:10.1021/acs.inorgchem.5c05828)
Supplement: Supplementary file 1 [file ic5c05828_si_001.pdf]

Supporting Information

Mixed Phenanthroline-Bipyridine Europium and Ytterbium Cryptates – Bright Luminophores with Small Radiative Lifetimes

Tobias Haas,† Timo Neumann,† Nicholas Jobbitt,‡ Xiaoyu Yang,‡ Hartmut Schubert,† David Hunger,‡,§ Michael Seitz*,†

† Institute of Inorganic Chemistry, University of Tübingen, Auf der Morgenstelle 18, 72076 Tübingen, Germany

‡ Institute of Physics (PHI), Karlsruhe Institute of Technology, 76131 Karlsruhe, Germany

§ Institute for Quantum Materials and Technologies (IQMT), Karlsruhe Institute of Technology, 76131 Karlsruhe, Germany

Email: michael.seitz@uni-tuebingen.de

Table of Contents

1. ^{1}H NMR Spectra S2

2. HPLC S4

3. Extended Photophysical Data S10

4. Crystallographic Data for **6-Na** S12

5. Details for the Calculation of τ_{rad} for **6-Yb** and **[D$_8$]-6-Yb** S13

6. References S14

1. ^{1}H NMR Spectra

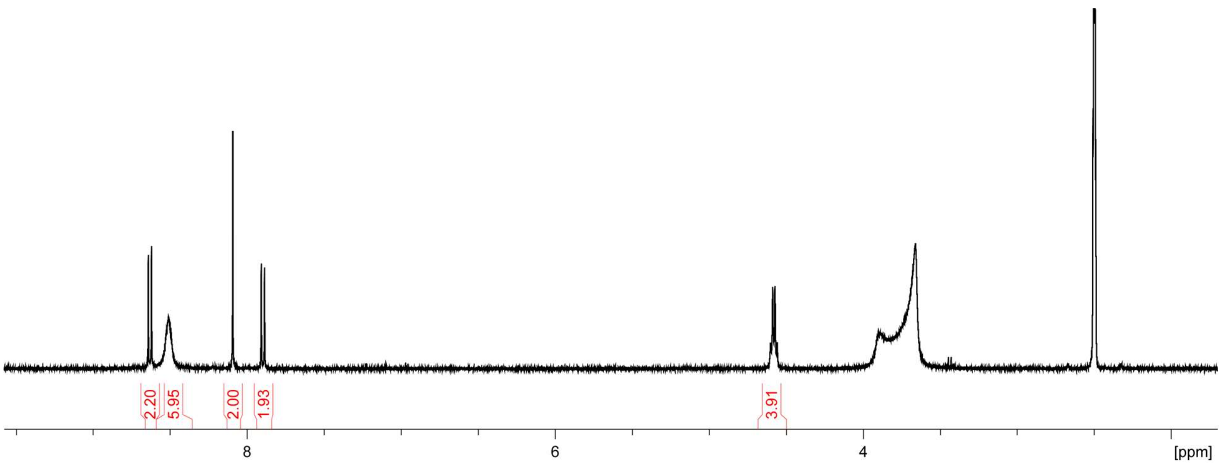

Figure S1. ^{1}H NMR spectrum (400 MHz, [D$_6$]-DMSO) of 2,9-bis(aminomethyl)-1,10-phenanthroline-trihydrobromide (**7**).

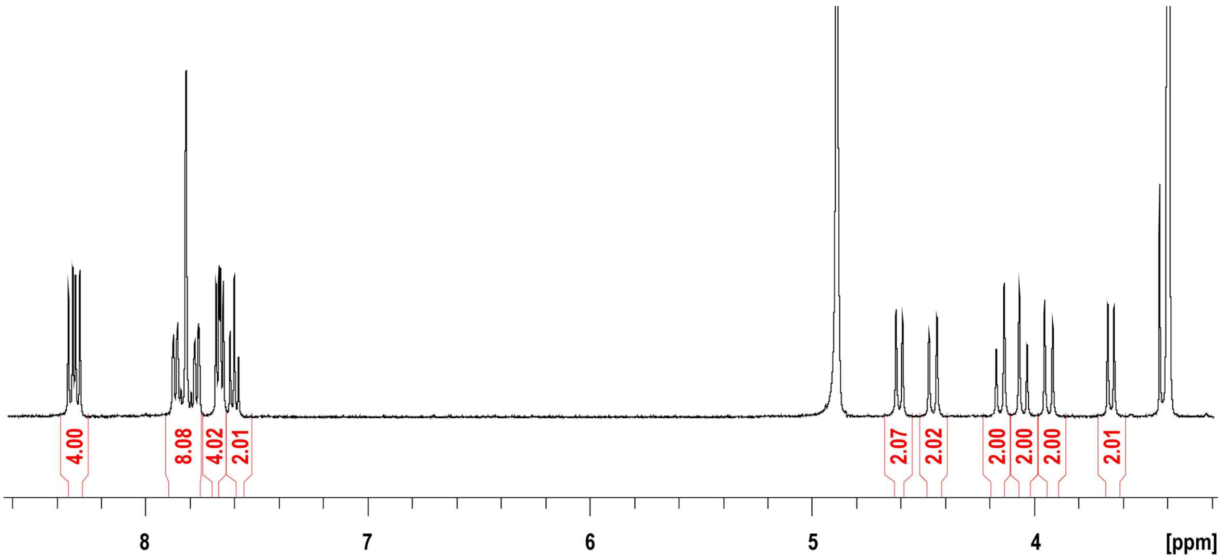

Figure S2. ^{1}H NMR spectrum (400 MHz, CD_3OD) of **5-Na**.

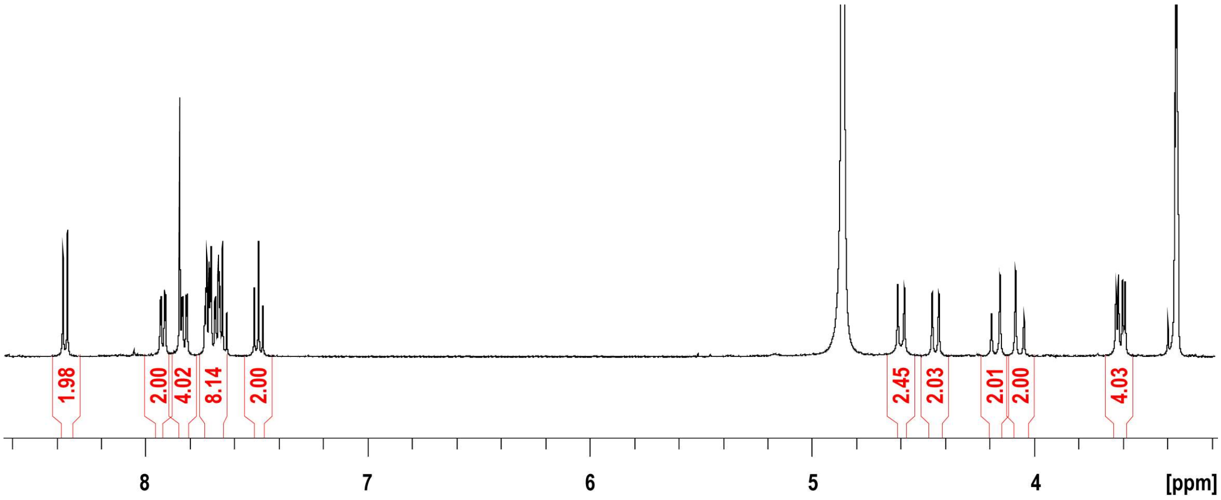

Figure S3. ^{1}H NMR spectrum (400 MHz, CD_3OD) of **6-Na**.

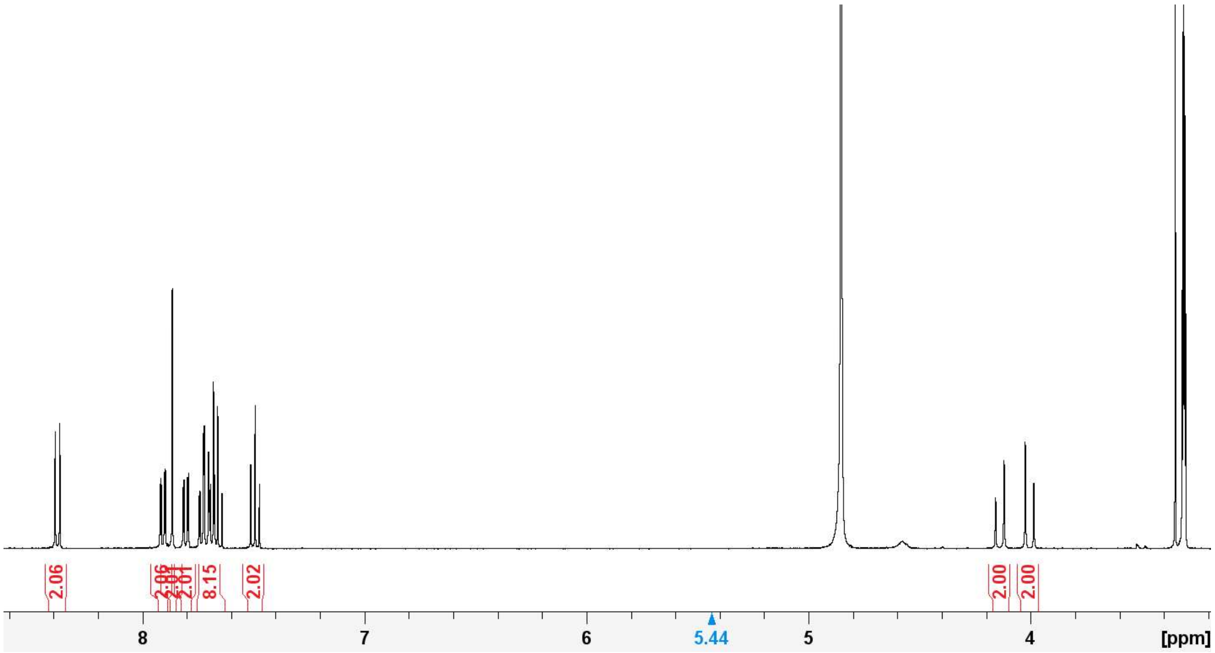

Figure S4. ^{1}H NMR spectrum (400 MHz, CD_3OD) of **[D$_8$]-6-Na**.

2. HPLC

Reversed-phase(RP)-HPLC was performed using Lichrospher RP-18e columns (Merck, semi-preparative: 250 mm×10 mm, 10 µm particle size; analytical: 125 mm×4 mm, 5 µm particle size) on a Knauer AZURA P6.1L system. The purity of the isolated fractions of the preparative separations was checked by subsequent analytical runs. All samples were dissolved in H_2O (HPLC grade) and the resulting solutions were filtered with a nylon membrane filter (GE Healthcare Life Sciences, 0.45 µm pore size) in a stainless-steel cartridge before injection. Flow rates of 3.0 mL/min and 1.0 mL/min were used for semi-preparative and analytical runs, respectively. The retention times of compounds naturally differ between analytical and semi-preparative conditions due to the different column dimensions and particle sizes. The cryptates were detected by UV absorption at λ = 300 nm. Using mobile phases A (degassed HPLC-grade H_2O + 1 vol.-% HPLC-grade CF_3COOH) and B (degassed HPLC-grade CH_3CN), the following programs were applied:

Analytical runs (flow rate 1.0 mL/min):

time [min]	%A	%B
0	85	15
5	85	15
19	45	55
25	45	55
40	85	15
50	85	15

Semi-preparative runs (flow rate 3.0 mL/min):

I (for Lu cryptates)			II (for Eu cryptates)			III (for Yb cryptates)		
time [min]	%A	%B	time [min]	%A	%B	time [min]	%A	%B
0	85	15	0	85	15	0	85	15
5	85	15	4	85	15	13	85	15
19	45	55	20	55	55	39	60	40
25	45	55	21	55	15	42	60	40
40	85	15	32	85	15	55	85	15
50	85	15	38	85	15			

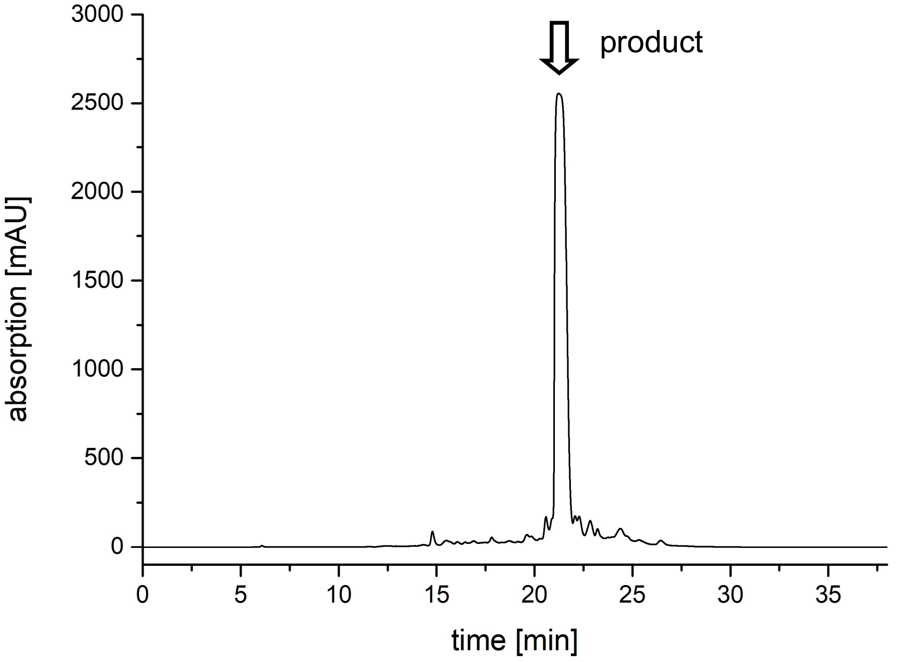

Figure S5. Semi-preparative HPLC trace for crude **5-Eu** (HPLC program II).

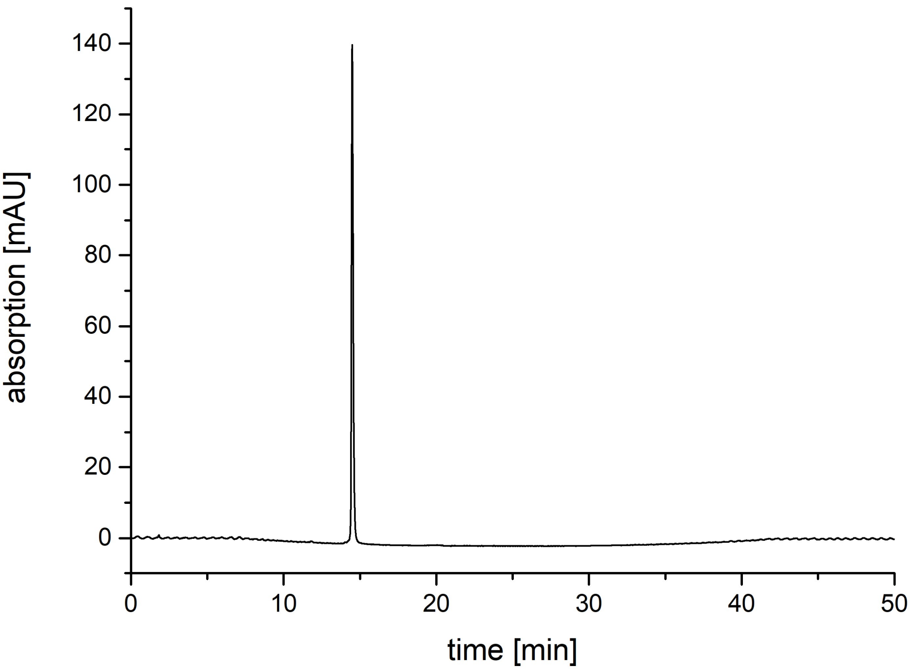

Figure S6. Analytical HPLC trace for purified **5-Eu** (after semi-preparative HPLC, see Figure S5).

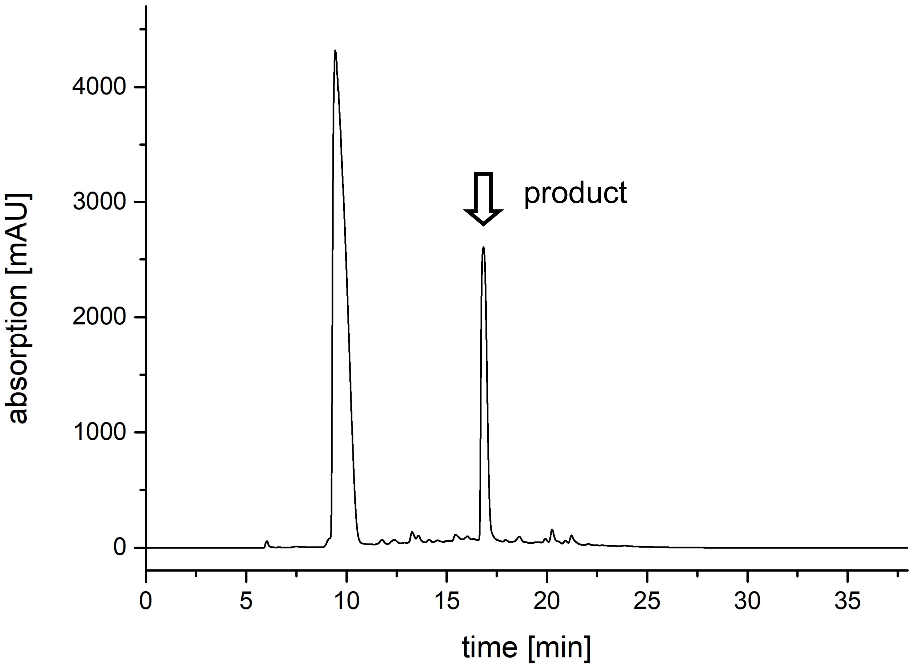

Figure S7. Semi-preparative HPLC trace for crude **6-Eu** (HPLC program II).

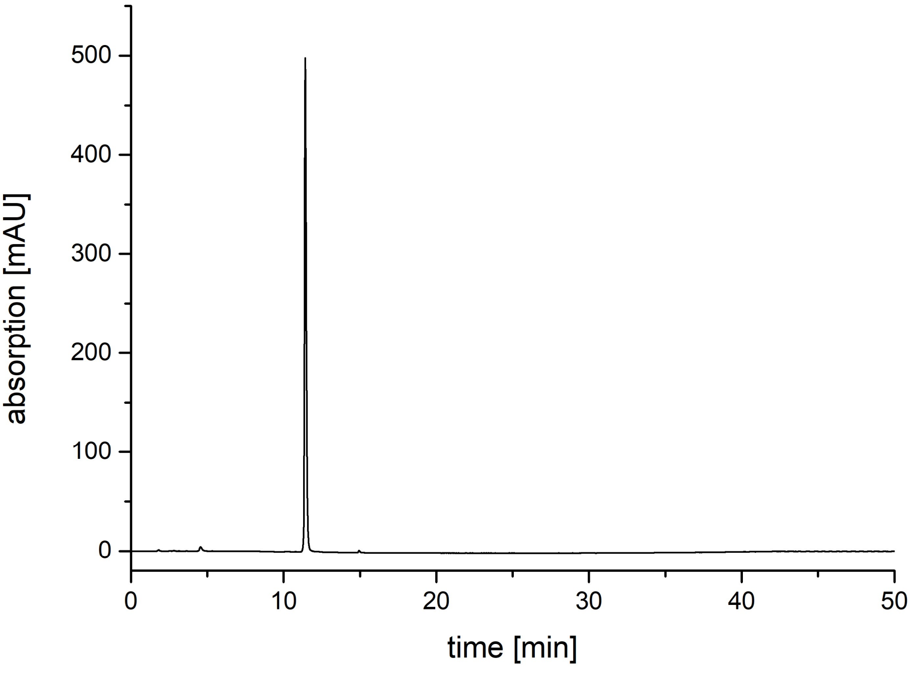

Figure S8. Analytical HPLC trace for purified **6-Eu** (after semi-preparative HPLC, see Figure S7).

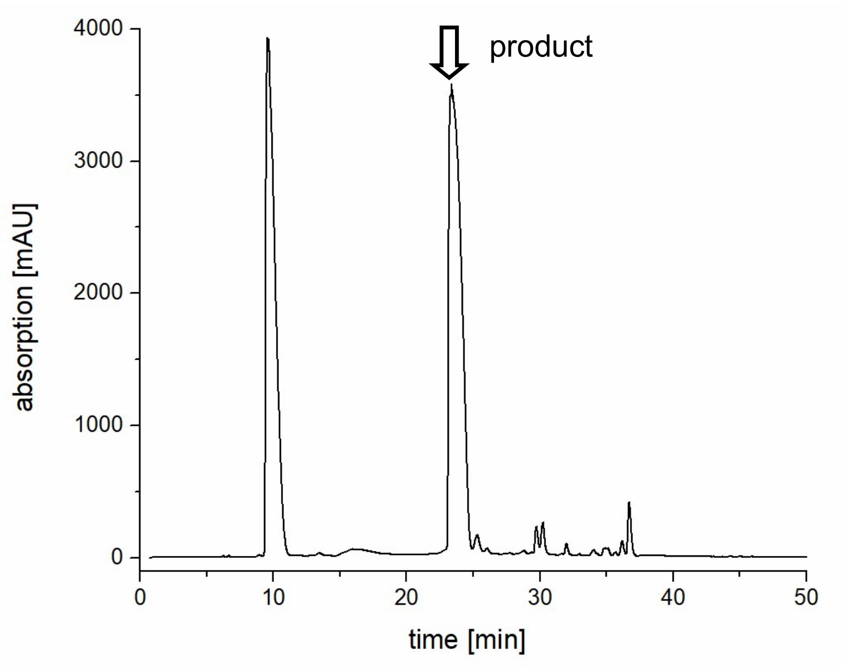

Figure S9. Semi-preparative HPLC trace for crude **6-Yb** (HPLC program III).

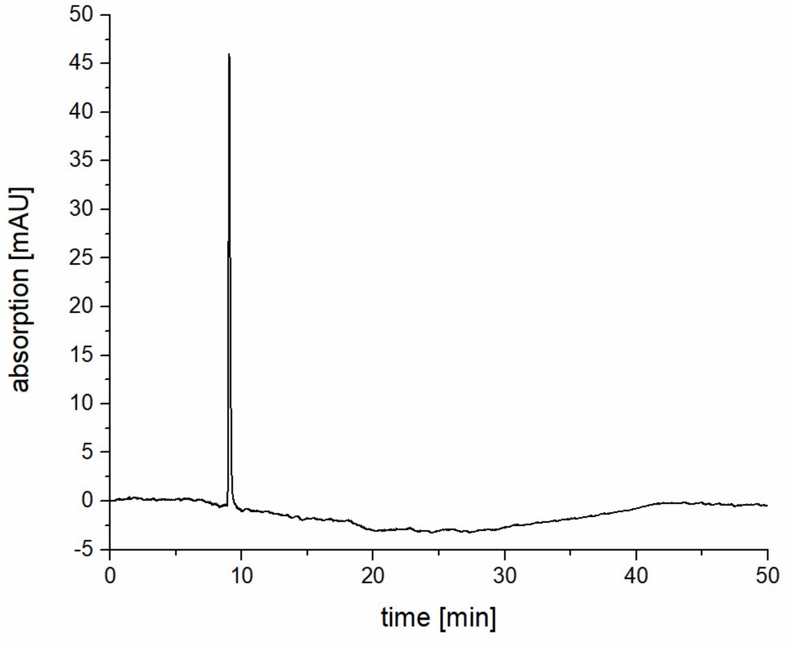

Figure S10. Analytical HPLC trace for purified **6-Yb** (after semi-preparative HPLC, see Figure S9).

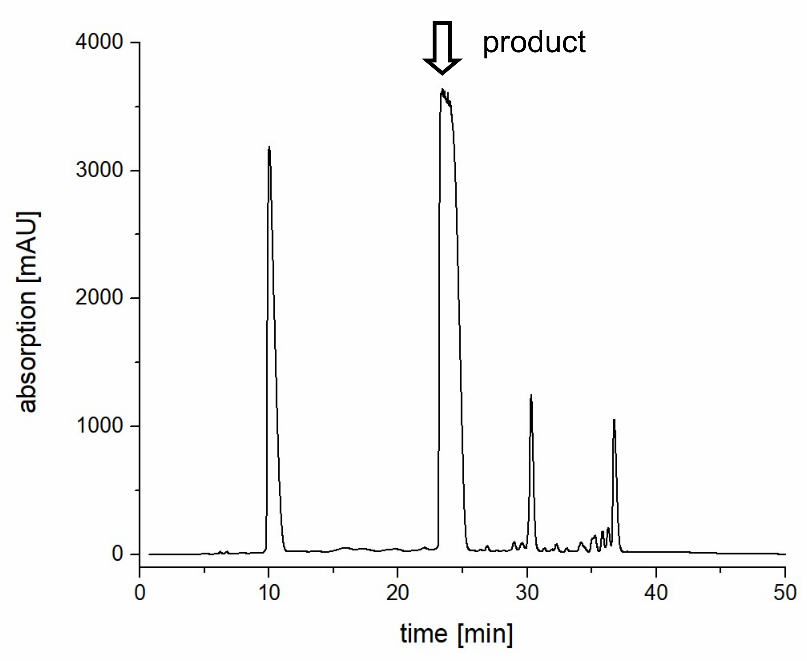

Figure S11. Semi-preparative HPLC trace for crude **[D$_8$]-6-Yb** (HPLC program III).

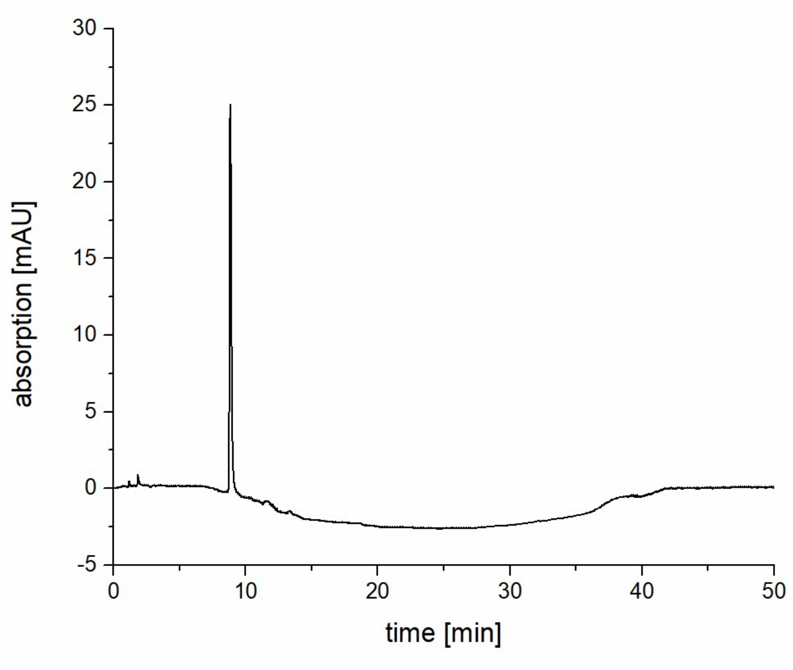

Figure S12. Analytical HPLC trace for purified **[D$_8$]-6-Yb** (after semi-preparative HPLC, see Figure S11).

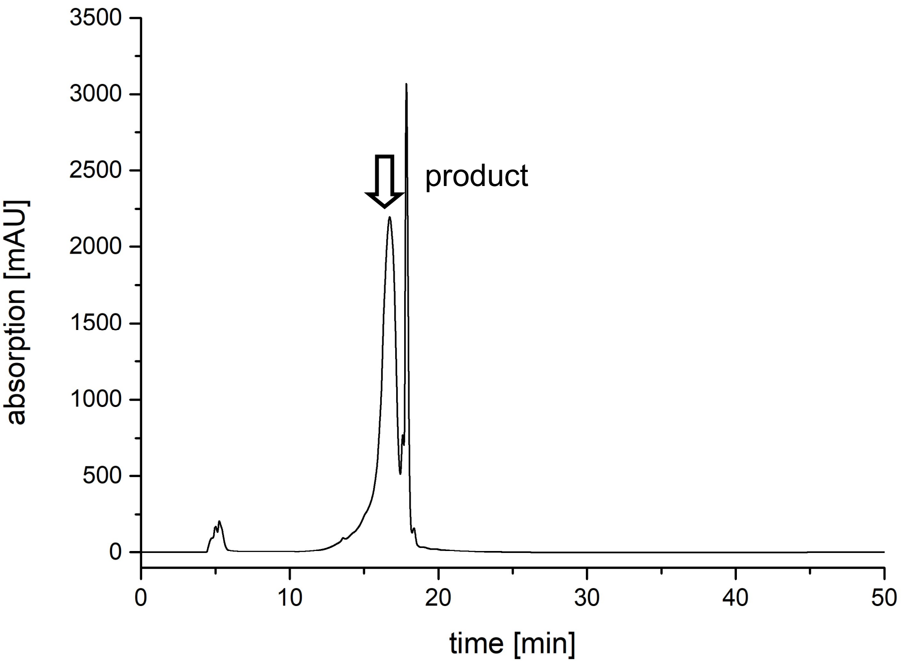

Figure S13. Semi-preparative HPLC trace for **5-Lu** (HPLC program I).

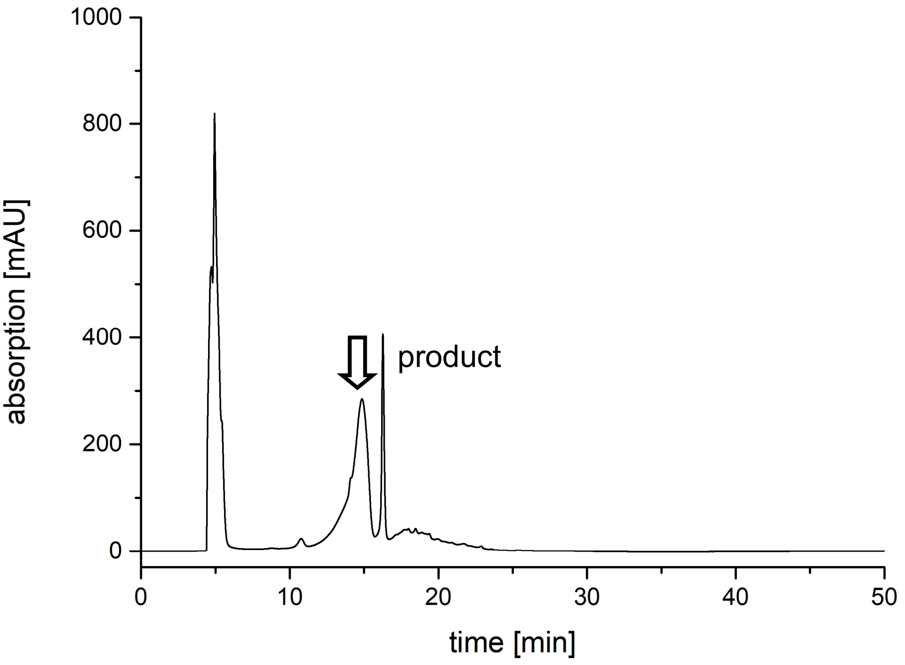

Figure S14. Semi-preparative HPLC trace for **6-Lu** (HPLC program I).

3. Extended Photophysical Data

3.1 UV Absorption Spectroscopy

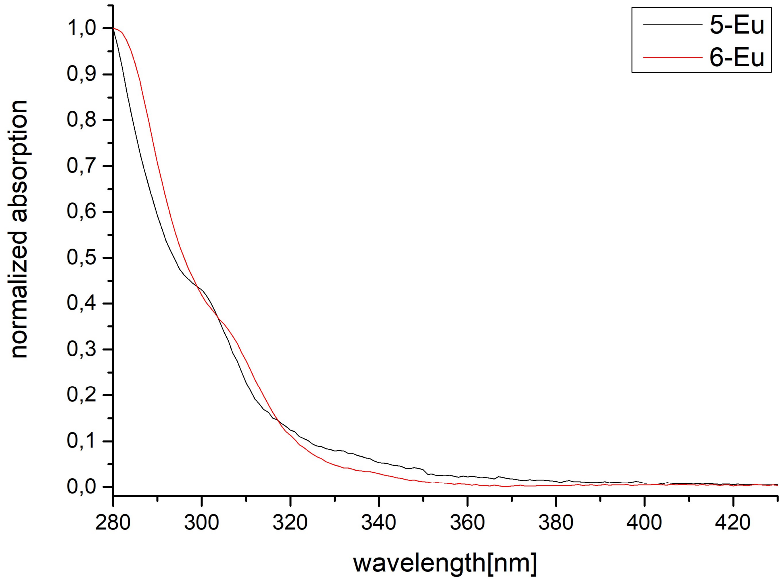

Figure S15. Normalized UV/vis absorption spectra of **5-Eu** (black) and **6-Eu** (red) in D_2O.

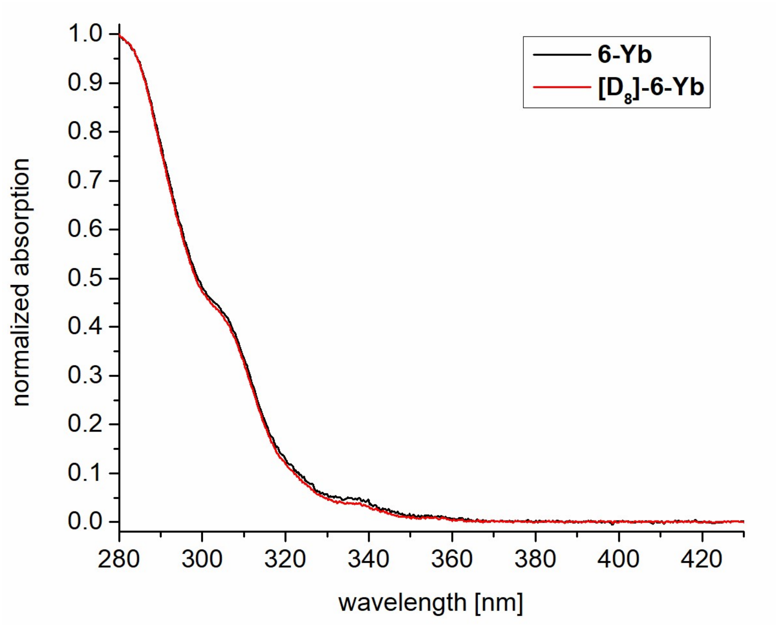

Figure S16. Normalized UV/vis absorption spectra of **6-Yb** (black) and **[D8]-6-Yb** (red) in CD_3OD.

3.2 Low Temperature Emission Spectra

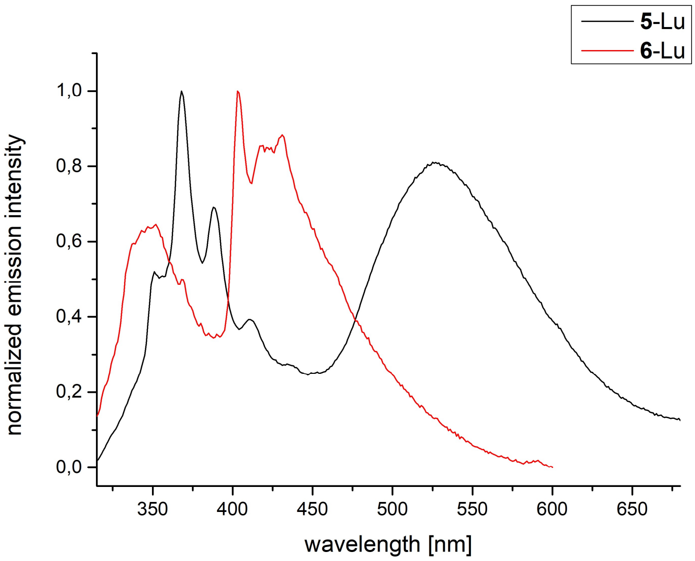

Figure S17. Low temperature emission spectra (λ_{exc} = 305 nm, T = 77 K) of the cryptates **5-Lu** (black) and **6-Lu** (red) measured in a CH_3OH/EtOH glass matrix (1:1, v/v).

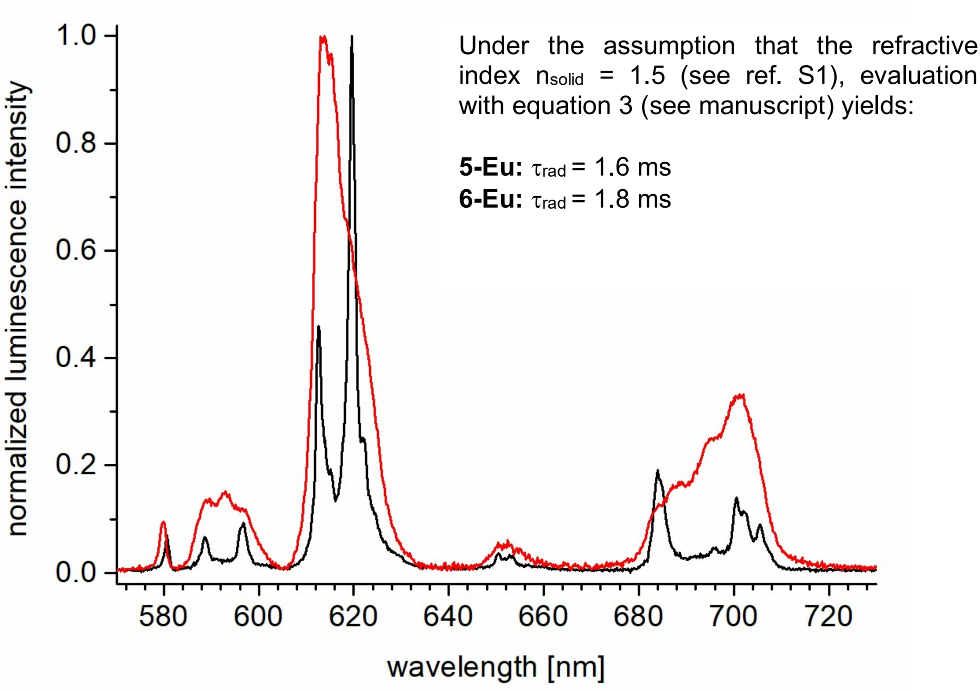

Figure S18. Steady-state emission spectra (powder, 298 K, λ_{exc} = 532 nm) for **5-Eu** (black) and **6-Eu** (red).

4. Crystallographic Data for 6-Na

Single crystals of **6-Na** were obtained by slow evaporation of a dichloromethane solution at room temperature. X-ray data were collected with a Bruker Smart APEX II diffractometer with graphite-monochromated Mo K_{α} radiation or a Bruker APEX II Duo diffractometer with a Mo IμS microfocus tube and a TRIUMPH monochromator. The programs used were Bruker's APEX2, including SADABS for absorption correction and SAINT for data reduction.[S2] The structure was solved with SHELXTL-2014 using direct methods and expanded using Fourier techniques (SHELXL-2018).[S3] The hydrogen atoms attached to carbon atoms were positioned geometrically but not refined. All such hydrogen atoms were constrained to ride on their parent carbon atoms. U_{iso}(H) values were set at 1.2 times U_{eq}(C).

Table S1. Selected crystallographic data for **6-Na**.

Empirical formula	$C_{38}H_{30}$
M_r [g mol^{-1}]	869.72
Crystal appearance	colorless prism
T [K]	100(2)
λ [Å]	0.71073 (Mo K_{α})
Crystal system	triclinic
Space group	P-1
Z	2
a [Å]	9.4159(4)
b [Å]	14.1304(5)
c [Å]	16.2863(5)
α [°]	92.061(2)
β [°]	101.977(2)
γ [°]	109.084(2)
V [Å^3]	1990.56(13)
D_{calc} [g cm^{-3}]	1.451
μ [mm^{-1}]	1.107
F(000)	900
Crystal size [mm × mm × mm]	0.21 × 0.20 × 0.18
θ range [°]	1.286-27.127
Limiting indices	-11 ≤ h ≤ 11<br>-18 ≤ k ≤ 18<br>-20 ≤ l ≤ 20
Collected refl.	39521
Independent refl.	8609
Absorption correction	empirical
Transmission (max., min.)	0.7455, 0.5696
Parameters/restraints	551 / 0
R_1 [I > 2σ(I)][a]	0.0433
wR_2 [I > 2σ(I)][b]	0.1069
R_1 (all data)[a]	0.0563
wR_2 (all data)[b]	0.1123
GOF on F^2	1.098
$\Delta\rho_{max,min}$ [e Å^{-3}]	0.525/-0.690

[a] R_1 factor definition: $R_1 = \Sigma (||F_0| - |F_c||) / \Sigma |F_0|$
[b] wR_2 factor definition: $wR_2 = [\Sigma w(F_0^2 - F_c^2)^2 / \Sigma w(F_0^2)]^{1/2}$. Weighting scheme: $w = 1 / [\sigma^2(F_0)^2 + (np)^2]$, $p = [F_0^2 + 2 F_c^2] / 3$

5. Details for the Calculation of τ_{rad} for 6-Yb and [D8]-6-Yb

Under the assumption that the 2J+1 sublevels of the ytterbium-centered states are really degenerate (or at least have the same population), the radiative lifetimes τ_{rad} of the *f-f* transition $^2F_{5/2}\rightarrow{}^2F_{7/2}$ in **6-Yb** and **[D$_8$]-6-Yb** can be calculated from the quantitative *f-f*-absorption spectrum ($^2F_{7/2}\rightarrow{}^2F_{5/2}$) with the following equation:[S4]

$$\frac{1}{\tau_{rad}} = 2303 \cdot \frac{8\pi c n^2 \cdot \tilde{\nu}_m{}^2}{N_A} \cdot \frac{(2J_l+1)}{(2J_u+1)} \cdot \int \varepsilon(\tilde{\nu})d\tilde{\nu} \qquad \text{with } \tilde{\nu}_m = \frac{\int \tilde{\nu}\cdot\varepsilon(\tilde{\nu})d\tilde{\nu}}{\int \varepsilon(\tilde{\nu})d\tilde{\nu}}$$

where the expressions have the usual meaning listed below:

c	speed of light (in cm s^{-1})
N_A	Avogardo's constant
n	refractive index (CD_3OD: n = 1.326)
$\tilde{\nu}_m$	barycenter of the transition ($^2F_{7/2}\rightarrow{}^2F_{5/2}$) as defined above
$2J_l+1$	degeneracy of the lower (ground) state (here for Yb: J_l = 7/2)
$2J_u+1$	degeneracy of the upper (excited) state (here for Yb: J_u = 5/2)
$\varepsilon(\tilde{\nu})$	molar extinction coefficient (in units of [M^{-1}cm^{-1}]) of the transition ($^2F_{7/2}\rightarrow{}^2F_{5/2}$) vs. the wavenumber $\tilde{\nu}$

6. References

[S1] Bünzli, J.-C. G.; Chauvin, A.-S.; Kim, H. K.; Deiters, E.; Eliseeva, S. V.; Lanthanide luminescence efficiency in eight- and nine-coordinate complexes: Role of the radiative lifetime. *Coord. Chem. Rev.* **2010**, *254*, 2623.

[S2] a) *Bruker AXS Inc. Madison, Wisconsin, USA,* 2007; b) G. M. Sheldrick, *SADABS, University of Göttingen, Germany,* 2008.

[S3] a) Sheldrick, G. M.; A short history of *SHELX*. *Acta Crystallogr. A* **2008**, *64*, 112; b) Sheldrick, G. M.; Crystal structure refinement with SHELXL. *Acta Cryst. Sect. C* **2015**, *71*, 3.

[S4] Werts, M. H. V.; Jukes, R. T. F.; Verhoeven, J. W.; The emission spectrum and the radiative lifetime of Eu^{3+} in luminescent lanthanide complexes. *Phys. Chem. Chem. Phys.* **2002**, *4*, 1542.
